# Biogeographic and metabolic studies support a glacial radiation hypothesis during *Chrysanthemum* evolution

**DOI:** 10.1093/hr/uhac153

**Published:** 2022-07-06

**Authors:** Xi Chen, Haibin Wang, Jiafu Jiang, Yifan Jiang, Wanbo Zhang, Fadi Chen

**Affiliations:** State Key Laboratory of Crop Genetics and Germplasm Enhancement, Key Laboratory of Landscaping, Ministry of Agriculture and Rural Affairs, Key Laboratory of Biology of Ornamental Plants in East China, National Forestry and Grassland Administration, College of Horticulture, Nanjing Agricultural University, 210095 Nanjing, China; College of Agriculture and Biological Sciences, Dali University, 671003 Dali, China; State Key Laboratory of Crop Genetics and Germplasm Enhancement, Key Laboratory of Landscaping, Ministry of Agriculture and Rural Affairs, Key Laboratory of Biology of Ornamental Plants in East China, National Forestry and Grassland Administration, College of Horticulture, Nanjing Agricultural University, 210095 Nanjing, China; State Key Laboratory of Crop Genetics and Germplasm Enhancement, Key Laboratory of Landscaping, Ministry of Agriculture and Rural Affairs, Key Laboratory of Biology of Ornamental Plants in East China, National Forestry and Grassland Administration, College of Horticulture, Nanjing Agricultural University, 210095 Nanjing, China; State Key Laboratory of Crop Genetics and Germplasm Enhancement, Key Laboratory of Landscaping, Ministry of Agriculture and Rural Affairs, Key Laboratory of Biology of Ornamental Plants in East China, National Forestry and Grassland Administration, College of Horticulture, Nanjing Agricultural University, 210095 Nanjing, China; State Key Laboratory of Crop Genetics and Germplasm Enhancement, Key Laboratory of Landscaping, Ministry of Agriculture and Rural Affairs, Key Laboratory of Biology of Ornamental Plants in East China, National Forestry and Grassland Administration, College of Horticulture, Nanjing Agricultural University, 210095 Nanjing, China; State Key Laboratory of Crop Genetics and Germplasm Enhancement, Key Laboratory of Landscaping, Ministry of Agriculture and Rural Affairs, Key Laboratory of Biology of Ornamental Plants in East China, National Forestry and Grassland Administration, College of Horticulture, Nanjing Agricultural University, 210095 Nanjing, China

## Abstract

Chrysanthemum (*Chrysanthemum morifolium* Ramat.) is an economically important plant species growing worldwide. However, its origin, especially as revealed by biogeographic and metabolomics research, remains unclear. To understand the geographic distribution of species diversity and metabolomics in three genera (*Chrysanthemum*, *Ajania*, and *Phaeostigma*), geographic information systems and gas chromatography–mass spectrometry were used in 19, 15, and 4 species respectively. China and Japan were two potential panbiogeographic nodes and diverse hotspots of *Chrysanthemum*, with species richness ratios of 58.97 and 33.33%. We studied different species from two hotspots which in similar geographical environments had closer chemotaxonomic relationships under the same cultivation conditions based on a cluster of 30 secondary metabolites. The average distribution altitude (ADA) differed significantly among *Chrysanthemum*, *Ajania*, and *Phaeostigma* in which it was 1227.49, 2400.12, and 3760.53 m.a.s.l. respectively, and the presence/absence of ray florets (RF) was significantly correlated with ADA (−0.62). Mountain landform was an important contributor to global *Chrysanthemum* diversity, playing a key role in the divergence and distribution pattern of *Chrysanthemum* and its allies. The Hengduan Mountains–Qinling Mountains (HDQ) in China was a potential secondary radiation and evolution center of *Chrysanthemum* and its related genera in the world. During the Quaternary glacial–interglacial cycles, this region became their refuge, and they radiated and evolved from this center.

## Introduction

Chrysanthemum (*Chrysanthemum morifolium* Ramat.) is an important ornamental plant whose origin has been explored continuously for many years [1]. However, its origin remains to be fully elucidated. Chrysanthemum was first cultivated in China [2, 3], and it likely originated from the natural or artificial hybridization and long-term artificial breeding of different wild *Chrysanthemum* species [4]. Evolutionary trajectories and migration histories of this genus and its allies play a vital role in the origin of chrysanthemum.

The colors of ray florets of cultivated chrysanthemum are very rich. However, they are relatively few in wild *Chrysanthemum* species, in which they are mainly yellow or white–purple. The yellow species are classified as the *C. indicum* group, while the white–purple species belong to the *C. zawadskii* group [5]. In addition, the *C. makinoi* group (species with white ray florets, including *C. rhombifolium* and *C. crissum*) is more closely related to the *C. indicum* group than the *C. zawadskii* group. However, gene flow between these groups needs to break various barriers, the most important of which is geographic isolation. Therefore, the origin and evolution of *Chrysanthemum* and its allies, especially in biogeography research [6–8], are key factors in exploring the origin of chrysanthemum.

Reconstruction of historical biogeography suggested the origin of *Chrysanthemum* in Central Asia, followed by eastward migration [9]. The relatively widespread tetraploid form of *C. indicum* expanded its range southward in the Pleistocene in China, most likely during the recent or previous glacial period [10]. However, *Chrysanthemum* has many members and is widely distributed globally. Although most members of *Chrysanthemum* are distributed in East Asia, some species are still distributed in South Asia, Europe, and North America. It is difficult to fully explain the geographic distribution pattern and migration trajectory of these species as a result of only eastward or southward migration. Biogeographic research on *Chrysanthemum* and its related species is largely lacking, especially in global and quantitative investigations.

Previous phylogenetic studies based on different genes have shown that many members of *Chrysanthemum* and *Ajania* are chimeric [11–14]. From the perspective of molecular phylogeny, it has been difficult to identify the main factors leading to the divergence of key traits, such as ray florets and tubular florets, especially environmental, geological, historical, and geographical factors [15], the effects of which have not been clearly explained.

Environmental selection is the core driving force of biological evolution [16]; not only is it reflected in the divergence of genes and traits, but it also has a significant impact on secondary metabolites [17, 18]. Plants synthesize hundreds of thousands of structurally diverse and lineage-, tissue-, or cell type-specific specialized metabolites [19], and there are myriad taxonomically restricted specialized metabolites [20]. Metabolomics provides multiple perspectives and strong evidence for the phylogeny and evolution of Asteraceae species, such as Lychnophorinae [21] and *Vernonia* [22]. The leaves and capitula of *Chrysanthemum* and its related groups often have a special aromatic smell and volatile components [23, 24]. These volatile secondary metabolites are usually used in the study of the essential oils, drug composition, and flavor for single species [25–29], where the materials are often dried or subjected to other treatments. However, there is a lack of research on the corresponding evolutionary relationships, in particular non-targeted metabolomics research on untreated fresh samples based on gas chromatography–mass spectrometry (GC–MS).

Here, through a large number of studies and specimen comparisons, as well as field investigations, the quantitative biogeography of *Chrysanthemum*, *Ajania*, and *Phaeostigma* was explored based on geographic information systems (GIS). The global diversity hotspots, potential origin centers, and migration paths are discussed. At the same time, the main factors influencing the differentiation of key traits are analyzed by combining the traits with environmental factors, such as altitude and annual average rainfall. Finally, non-targeted metabolomics research was carried out, and the evolution of key taxonomic traits and potential development history are comprehensively analyzed in combination with morphological traits. In this study, we aimed to provide novel perspectives and evidence of the evolutionary trajectories and migration histories of *Chrysanthemum* and its allies.

**Figure 1:**
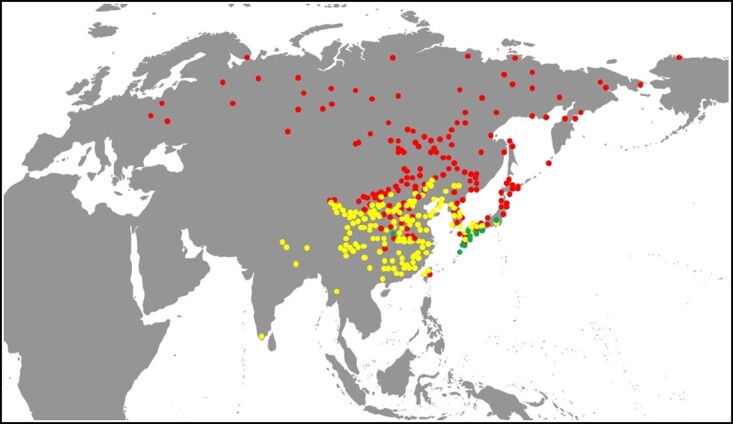
Distributions of the *C. indicum* group (yellow), *C. makinoi* group (green), and *C. zawadstii* group (red) of *Chrysanthemum*.

## Results

### Taxa and distribution of *Chrysanthemum* and its allies

Based on the statistics and identification of herbaria and documentations, as well as field investigations, 39 species of *Chrysanthemum* were identified in terms of geographic information record (besides 24 subspecies, varieties, and forms). There were 37 *Ajania* species and seven species of *Phaeostigma* worldwide (Supplementary Data Table S2). It should be emphasized that *C. morifolium* Ramat. is listed in the genus *Chrysanthemum* in many studies [30–32]. Geographically, it has been artificially spread worldwide. Therefore, cultivated chrysanthemum, artificial hybrids, and cultivars were not among the 39 species in this study. In addition, the geographic distribution, altitude, habitat, herbaria, and references of each species were determined (Supplementary Data Table S2, Supplementary Data Fig. S1).

The distribution of the three genera was affected by the climate and vegetation types (Supplementary Data Figs S1 and S2). Generally, they showed a three-level transitional distribution pattern of *Chrysanthemum* (humid/semi-humid) – *Phaeostigma* (semi-humid/semi-arid) – *Ajania* (semi-arid/arid). A overlapped distribution was observed in the transition region and boundary area of the different climate types (Supplementary Data Fig. S2). The global distribution of *Chrysanthemum* and *Ajania* correlated with the rules of overall separation and local cross. The Himalayas and the region of southwest to northeast China were identified as a clear dividing line. *Chrysanthemum*, but not *Ajania*, was distributed in the southeastern part of the line, where the vegetation is good and the climate is humid (Supplementary Data Fig. S2).

**Figure 2:**
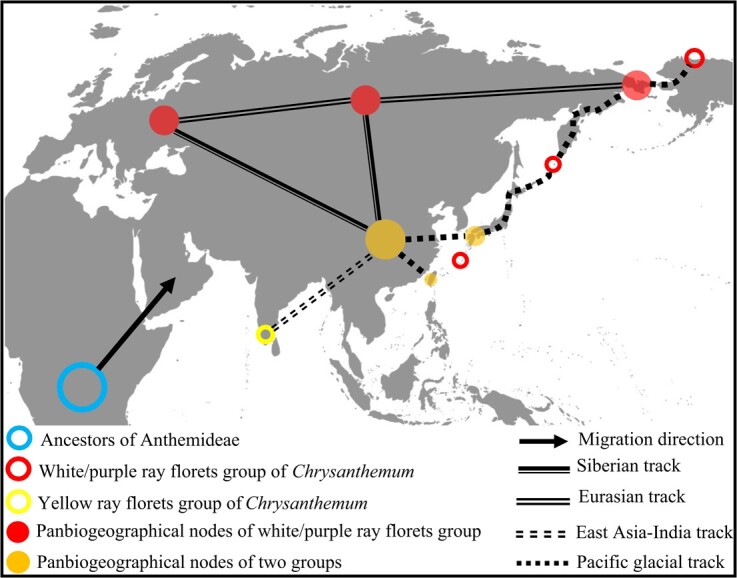
Generalized tracks of the global *Chrysanthemum* and potential panbiogeographic nodes.

**Table 1:** Species distribution and topographic features of diversity hotspots.

Hotspot	Species richness	Endemic species richness	Total species richness	Ratio of richness (%)	Area ratio between hotspot and total distribution area (%)	Sørenson index (SI)	Ratio of endemic species distributed in mountainous (%)	Ratio of endemic species distributed along the coast (%)	Ratio of endemic species distributed on small islands (%)	Ratio of species living distributed in stony/rocky habitats (%)
CSNMS (*C.*)	23	14	39	58.97	6.23	0.17	100.00	0.00	0.00	43.47
JMISI (*C.*)	13	7	39	33.33	1.23		42.65	57.14	28.57	46.15
CHNMS (*C.* + *A.* + *P.*)	59	37	83	71.08	6.28	0.10	100.00	0.00	0.00	49.15
JMISI (*C.* + *A.* + *P.*)	18	11	83	21.69	0.87		36.36	54.54	18.18	44.44

**Figure 3:**
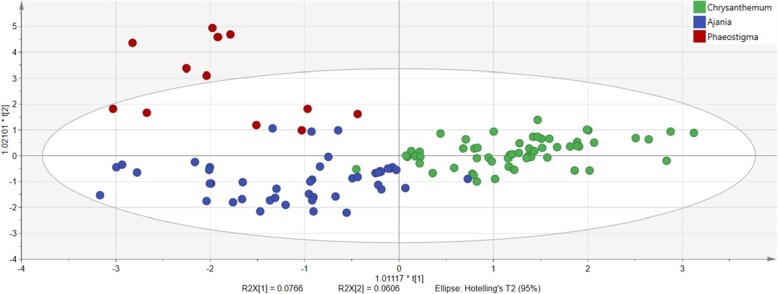
OPLS-DA of *Chrysanthemum*, *Ajania*, and *Phaeostigma* based on 30 secondary metabolites.

For the three groups of *Chrysanthemum*, the distribution of the *C. makinoi* group was relatively narrow: only in central China and south central Japan. The *C. indicum* group was mainly distributed in the middle and low latitudes of the southern extremity of the northern hemisphere (south of 45°N), while the *C. zawadstii* group was mainly distributed in the middle and high latitudes of the northern end of the northern hemisphere (north of 24°N). The three groups only crossed each other in China and Japan (Fig. 1). In addition, through field investigation, we found that the *C. indicum* and *C. zawadstii* groups of *Chrysanthemum* and their allies were distributions overlapped in central, north, and northeast China (Supplementary Data Fig. S3). This breaks the barrier caused by geographical isolation, and provides the possibility of gene flow among related species.

The southernmost species in the white-purple group were *Chrysanthemum morii* and *C. horaimontanum* from the Taiwan province of China, which formed a gap with other members of the group widely distributed at high latitudes. Discontinuity was also observed in the northwest corner of North America, Japan, and many islands from the western Pacific Ocean to the Eurasian continent (Fig. 2). However, the achenes of *Chrysanthemum* have no pappus and they are difficult to spread by wind or ocean currents. They are most likely to spread along land bridges and the continental shelf, which is the shortest and most effective path. We inferred that *Chrysanthemum* migrated and radiated between Eurasia and North America, as well as between East Asia and Taiwan, Japan, and other islands and their surrounding island chains, through land bridges and land formed by the sea-level decline during the Quaternary glacial period, i.e. the Pacific glacial track (Fig. 2). For the yellow group, the southernmost distribution area, southern India [Dindigul, *C. indicum*, K (001118855)] was discontinuous with the main distribution areas of China and its surrounding areas. The main geographic isolation obstacles were the Himalayas, the Hengduan Mountains of China, and the dense jungle of northern Myanmar (Fig. 1). Therefore, the shortest migration path was the East Asia–India track. As for the two groups, there were three nodes in the Central and Taiwan province of China and Japan (Fig. 2). Among them, the central region of China was the node with the most intersection tracks. Therefore, the central node of China plays a crucial role in the migration and evolution of *Chrysanthemum* in the world. It was not only an important node with the most intersection tracks but also the geographic center of the intersection of the two groups in the world. In addition, the distribution patterns and discontinuous areas of species such as *C. indicum* (Supplementary Data Fig. S1 a 14) and *C. zawadskii* (Supplementary Data Fig. S1 a 38) show that *Chrysanthemum* has experienced radiation in different directions and degrees during its evolution.

### Diversity hotspots of *Chrysanthemum* and its allies

There were two diversity hotspots of *Chrysanthemum*, including the southwest to northeast mountains group and surrounding areas in China (CSNMS), and the four major islands and surrounding islands of Japan (JMISI) (Table 1), with species richness ratios of 58.97 and 33.33%, respectively (Table 1). China had 14 endemic species, while Japan had 7; the Sørenson index (SI) between them was 0.17. For the three genera *Chrysanthemum*, *Ajania*, and *Phaeostigma*, there were also two hotspots in China and Japan, respectively, and the SI was very low (Table 1). Although the species similarity between different diversity hotspots was low, there were still some species distributed in two separate hotspots, such as *Chrysanthemum indicum*, *C. zawadskii*, *C. seticuspe*, and *Ajania pallasiana*. The migration and radiation of these species in different hotspots most likely occurred along the Pacific glacial track.

### Analysis of secondary metabolites

The taxa, number of peaks, and sum of peak areas based on GC–MS of 38 species from *Chrysanthemum*, *Ajania*, and *Phaeostigma* are shown in Supplementary Data Table S7. We found that, under the same cultivation conditions, the number of peaks and sum of peak areas of three genera showed significant differences, among which *Ajania* was the highest and *chrysanthemum* was the lowest (Supplementary Data Table S7, Supplementary Data Fig. S5). The 30 selected metabolites for PCA and cluster analysis and their Chemical Abstracts Service numbers (CAS) are shown in Supplementary Data Table S8. The results of PCA indicated that the 95% confidence interval of *Chrysanthemum* was completely chimeric in *Ajania*, and the vector distribution trends of the two confidence intervals were similar (Supplementary Data Fig. S4). From this point of view, the relationship was so close that it was difficult to distinguish them from each other. Although the confidence interval of *Phaeostigma* crossed with the former two, the proportion of crossed regions was not high, and the vector distribution of *Phaeostigma* showed a cross trend with that of *Chrysanthemum* and *Ajania*, not parallel. Therefore, the relationship between *Phaeostigma*, *Chrysanthemum*, and *Ajania* was relatively distant.

The results of OPLS-DA are shown in Fig. 3, in which *Chrysanthemum* and *Ajania* were separated as a whole. However, there was still a small cross, which made it difficult to completely separate them. *Phaeostigma* was completely separated from *Chrysanthemum* but crossed with *Ajania* in a small area. The results were in good agreement with those of PCA, i.e. the relationship between *Phaeostigma* and *Chrysanthemum* was relatively distant, while *Phaeostigma* and *Ajania* were close. *Ajania* is located in the transitional region between *Phaeostigma* and *Chrysanthemum*. The results of 200 permutations (Supplementary Data Fig. S10) for the OPLS-DA model indicated that the model was effective. However, the values of R2X, R2Y, and Q2 were <0.4, so the accuracy and stability of the model were relatively low. One of the reasons was that the secondary metabolites themselves were complex and diverse, and the correlation between metabolites was relatively low, resulting in the divergence of overall data between the three genera. Moreover, the ratio of the peak area of most metabolites to the internal standard was between 0 and 10. However, the ratio in some species of *Phaeostigma* and *Ajania* was too high, which led to a decrease in fitting stability.

**Figure 4:**
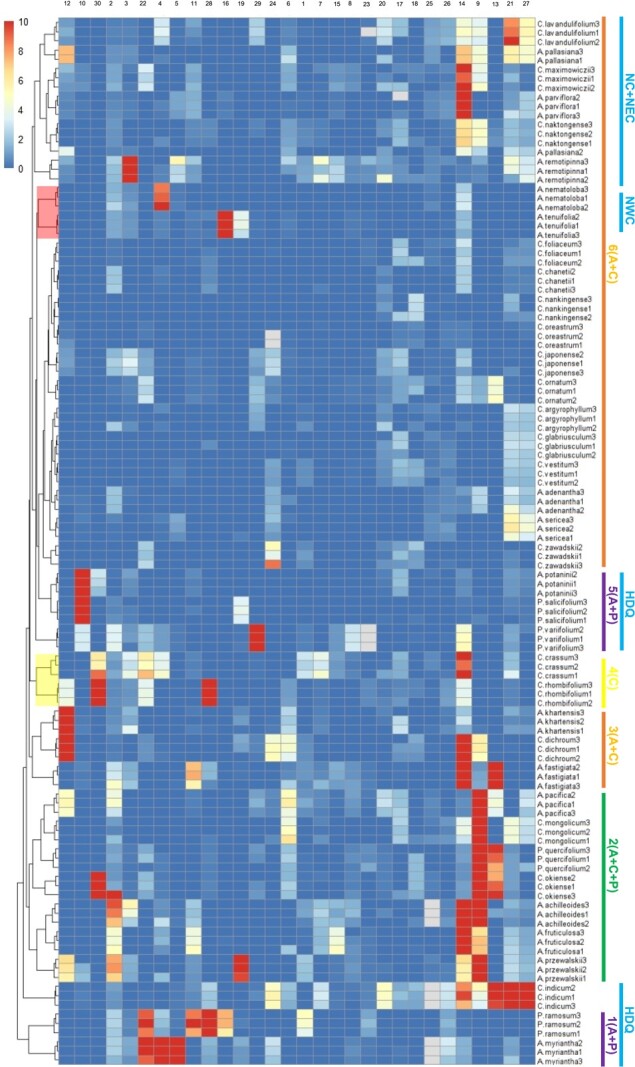
Cluster of *Chrysanthemum*, *Ajania*, and *Phaeostigma* based on 30 secondary metabolites. (1–6) Major branches. Blue line, geographical distribution of species: HDQ, Hengduan Mountains—Qinling Mountains; NC, North China; NEC, Northeast China; NWC, Northwest China; red branch, species with close or crossed geographic distribution areas; yellow branch, species with separated and distant geographic distribution areas. Secondary metabolites are as shown in Supplementary Data Table S8.

**Figure 5:**
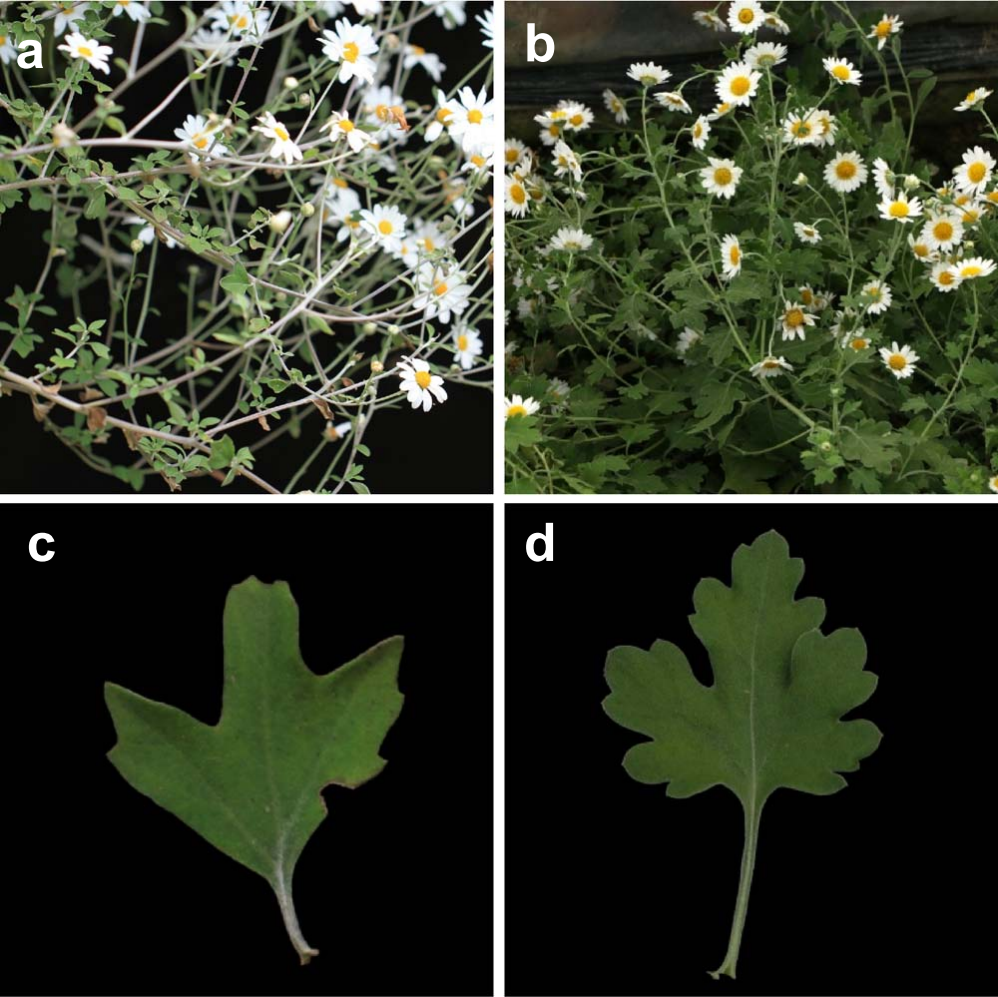
Phenotypic comparison of plant and leaf of (**a**, **c**) *Chrysanthemum rhombifolium* and (**b**, **d**) *Chysanthemum crassum*.

To prevent the overall data from being too divergent due to the high content of individual secondary metabolites, the data with the ratio of metabolite peak area to internal standard peak area ≥10 was selected as 10 in cluster analysis. There were 38 species, with a total of 114 samples, forming six main cluster clades: 1 (*Ajania* (A) + *Phaeostigma* (P)), 2 (A + *Chrysanthemum* (C) + P), 3 (A + C), 4 (C), 5 (A + P), and 6 (A + C) (Fig. 4). Among them, the six branches had the largest number of members, which were composed of 20 species of *Chrysanthemum* and *Ajania*, located at the top of the cluster. Other branches were composed of two or three genera. The bottom of the cluster was a branch composed of *Phaeostigma ramosum* and *Ajania myriantha*. We analyzed the traits of the two species in depth and found that many phenotypes of the two species were highly similar. First, both were members of the white–purple group, and their capitula were similar; second, the leaves of both plants were trisegmented, and the leaf shape, leaf texture, and indumentum were similar. The main difference was that the degree of leaf division in *Chrysanthemum crassum* was deeper (Fig. 5). The species in some clades showed a certain geographic distribution pattern; i.e. species with close or crossed geographic distribution areas tended to cluster into the same or relatively close clades, even if these species come from different genera. For example, the members of branches 5 and 1 were all from the same region, namely HDQ, while the top members of branch 1 were all from North and Northeast China (NC + NEC). In addition, some smaller branches also showed strong geographic distribution characteristics, such as *Ajania nematoloba* and *A. tenuifolia* (Fig. 4, red branch); they both came from Northwest China (NWC). The location of the two samples was very close, and the geographic environmvent was very similar.

### Topographic features of diversity hotspots

In order to explore the relationship between the above secondary metabolites and the morphology of different species between the two hotspots in China and Japan, as well as the reasons for diversity and species formation, we conducted a DEM-based topographic analysis. We found that the common geomorphological features of *Chrysanthemum* (CSNMS) and the three genera (CHNMS) diversity hotspots were the high mountains from the southwest to the northeast of China and their surrounding transition zones (Table 1), mainly the Hengduan Mountains, Qinling Mountains, Yinshan Mountains, Taihang Mountains, and Yanshan Mountains. The difference was that the CHNMS added the Himalayas into its range, and the regions with high diversity of the three genera were concentrated in the Hengduan Mountains–Qinling Mountains (HDQ), with the elevation of their main peaks being >3000 m.a.s.l.

The species distribution hotspots in Japan were mostly mountainous landforms (Table 1). The diversity of *Chrysanthemum* and *Ajania* was also greatly affected by mountainous topographic features, especially *Ajania*, which depended particularly on high mountains. Although some *Ajania* species, such as *A. pacifica*, *A. shiwogiku*, and *A. kinokumiense*, were distributed along the coast, they were still relatively close to the high mountains in central Japan. The main difference from the hotspots in China was that several endemic species in Japan were distributed on the coast or surrounding small archipelagoes, such as *C. crassum* (on Amami islands) and *C. okiense* (on the Oki Islands), which was consistent with the distribution of three endemic species in the Taiwan province of China. The geographical isolation of islands hindered the gene flow among related species to a great extent. Besides, these relatively isolated populations must face selection in harsher habitats to drive their rapid evolution. The result was reflected in the adaptation of traits and metabolites to the environment.

In addition, 92.31% of *Chrysanthemum* species were found to be distributed in mountains and their surrounding areas, accounting for only ~7.60% of the total distribution area in the world. The mountain landform is an important factor in speciation, and plays a key role in the evolution and distribution pattern of *Chrysanthemum*. Furthermore, according to our field investigation (Supplementary Data Figs S3 and S7) and document records (Supplementary Data Table S2), ~51.28, 45.95, and 71.43% of species in *Chrysanthemum*, *Ajania*, and *Phaeostigma* were related to stony and rocky topographies, which play a vital role in their survival, distribution, and speciation worldwide. This high adaptability to rocky habitats allows them to radiate rapidly in the dry environment during glacial periods.

**Figure 6:**
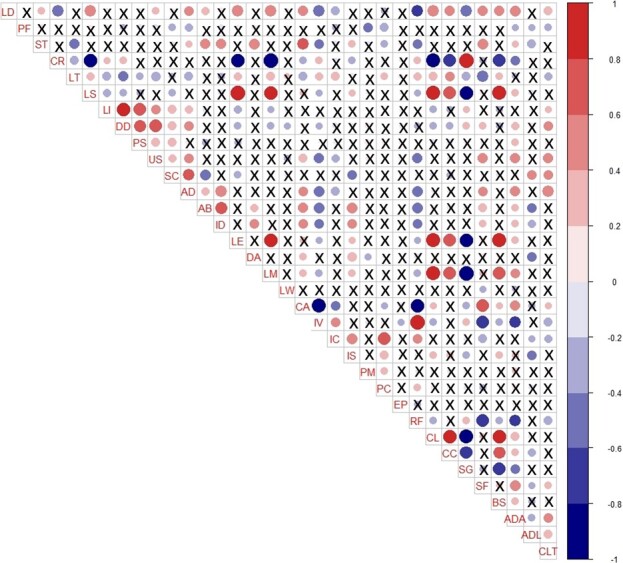
Correlation matrix for 31 traits and three environmental factors of *Chrysanthemum*, *Ajania*, and *Phaeostigma.* ‘×’ indicates non-significant coefficients at the α = 0.05 level. Expansions of abbreviations are shown in Supplementary Data Tables S5 and S6. Ray florets (RFs), the key characteristic distinguishing *Chrysanthemum* from *Ajania*, tended to disappear with increasing altitude (ADA) and decreasing annual average rainfall (CLT).

### Trait segregation among the three genera and environmental factors

Although the secondary metabolites of *Chrysanthemum* and its allies showed certain patterns with geographic environment and distribution, the special branch formed by *C. rhombifolium* and *C. crassum* indicated that there was also a certain relationship between secondary metabolites and traits (Fig. 4). Trait segregation is a critical factor in species formation and genus differentiation. Our PCA results based on 31 traits formed two principal components: PC1 (27.5%) and PC2 (19.5%). The 95% confidence intervals of *Chrysanthemum*, *Ajania*, and *Phaeostigma* showed a pattern of overall separation and partial superposition (Supplementary Data Fig. S6), most of which indicated that there was a large distance from *Chrysanthemum* and *Ajania* to *Phaeostigma* while *Ajania* was close to *Chrysanthemum.* In PC1, the traits with higher positive contribution rates were corolla colors of tubular florets (CC, 0.27), lateral expansion of petiole base (LE, 0.31) (Supplementary Data Fig. S8), leaf scar (LS, 0.31) (Supplementary Data Fig. S9), corolla lobes of tubular floret (CL, 0.32), leaf margin (LM, 0.31), brownish style-branches (BS, 0.29), and lignified degree (LD, 0.24). These were the main characteristics that distinguished *Phaeostigma* from *Ajania* and *Chrysanthemum*. In addition to the absence of ray florets (RF, −0.31), *Ajania* tended to be more densely compound-corymbose at the apices of branches, have deeper indumentum and leaf divisions, and more elegant ultimate segments than *Chrysanthemum*.

**Figure 7:**
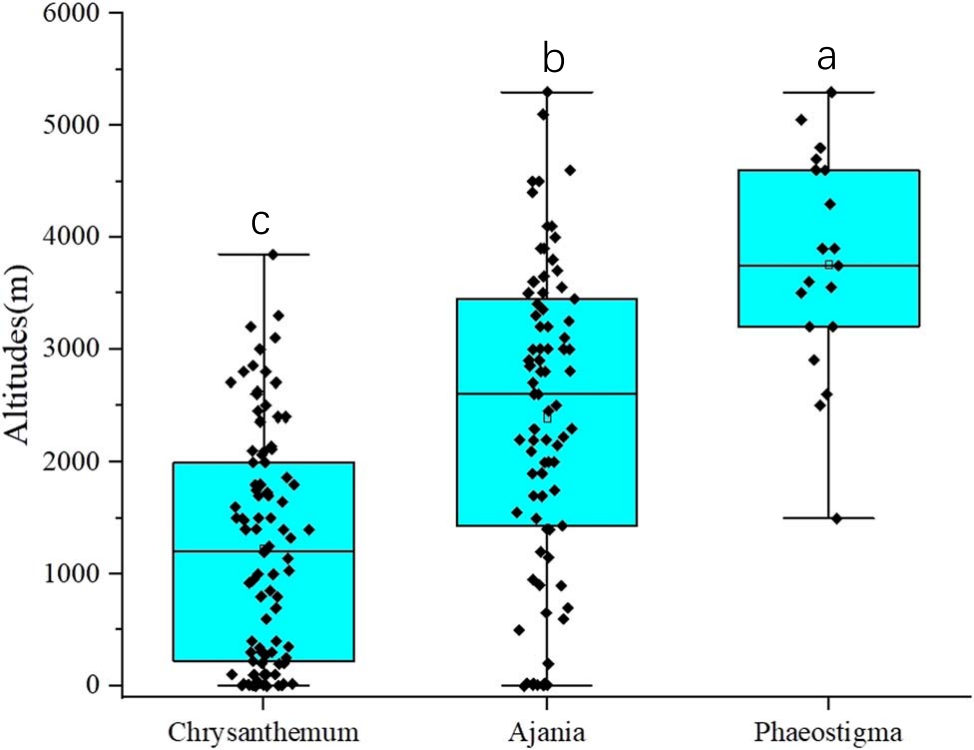
Distribution altitudes of *Chrysanthemum*, *Ajania*, and *Phaeostigma* (*P* < .05).

We found that, in addition to the topographic features, there were three factors—average distribution altitude (ADA), climate type (CLT, dominated by annual average rainfall), and average distribution latitude (ADL)—that affected the segregation of some important traits of *Chrysanthemum* and related groups. A total of 67.74, 48.39, and 35.48% of the traits were significantly correlated (α = 0.05) with ADA, ADL, and CLT, respectively (Fig. 6). In addition, many of the key traits used to define the relationship and important segregation among the three genera were significantly correlated (*P* < .05) with ADA, such as RF (−0.62), CL (0.35), involucres (IV, −0.63), LD (0.52), same color on bisexual floret and marginal female floret (SF) (0.51), ultimate segments (or cleft teeth) shapes (US) (0.50), capitula (CA) (0.49), leaf margin (LM) (0.42), indumentum (ID) (0.27), and CC (0.27). In addition, according to the previous field survey and document statistics, the average distribution altitude of *Chrysanthemum*, *Ajania*, and *Phaeostigma* was 1227.49, 2400.12, and 3760.53 m.a.s.l., respectively (P <05). The differences between the groups were >1000 m (Fig. 7). Therefore, the divergence among them was significantly affected by altitude.

CLT also affected these important traits, such as RF (−0.36), IV (−0.36), AD (0.60), US (0.51), SC (0.43), DD (0.43), LD (0.37), and SF (0.26). In contrast, latitude had little effect on these important traits. There were also significant correlations among the traits (α = 0.05), and some of the correlations were higher than .80. The statistical results showed that RFs, the key characteristic distinguishing *Chrysanthemum* from *Ajania*, tended to disappear with increasing altitude and decreasing annual average rainfall. The divergence of these key characteristics was directly related to the origin and evolutionary history of *Chrysanthemum* and its related species.

## Discussion

### Feasibility and stability of metabolomic(s) for phylogenetic and evolutionary research of *Chrysanthemum* and its allies

*Ajania* Poljakov (Anthemideae, Asteraceae) is a sister group closely related to *Chrysanthemum*, which can be distinguished by its disciform capitula; *Chrysanthemum*, on the other hand, has radiate capitula [33]. Based on the brownish style branches, erect corolla lobes, and microechinate pollen, Muldashev [48] separated *Phaeostigma* from *Ajania* [49]. However, Ohashi and Yonekura [50] included *Ajania* and *Phaeostigma* in *Chrysanthemum*, because hybrids between *Chrysanthemum* and *Ajania* are easy to obtain. Our results indicated that, in terms of OPLS-DA based on 30 secondary metabolites (Fig. 3), *Chrysanthemum* and *Ajania* were separated from each other as a whole. However, the results of PCA indicated that the 95% confidence interval of *Chrysanthemum* was completely chimeric in *Ajania*, and the vector distribution trend of the two confidence intervals was similar through metabolomics (Supplementary Data Fig. S4). Meanwhile, the relationship between *Phaeostigma* and *Chrysanthemum* and *Ajania* was relatively distant (Supplementary Data Fig. S4). Therefore, we were conservative about whether *Chrysanthemum* and *Ajania* should be merged into a single genus and become two subgenera of this genus.

Metabolites are often related to adaptation of plants, and thus are strongly subjected to natural selection, which may drive evolutionary convergence. Moreover, metabolic data are inherently noisy. In order to avoid the adverse effects caused by environmental conditions, all our materials were cultivated under the same conditions and sampled at the same growth stage and time as far as possible. We used liquid nitrogen to treat fresh materials for quenching enzyme activity, while ethyl acetate was used as the solvent and nonyl acetate as the internal standard for GC–MS.

This method detects many secondary metabolites. However, most of them are terpenoids, and it is difficult to find one or two terpenoids that can be used as taxonomically restricted specialized metabolites between genera to define all members of *Chrysanthemum*, *Ajania*, and *Phaeostigma*. Besides, due to the high content of some metabolites the interval was large for *Phaeostigma*, resulting in reduced model stability of OPLS-DA. In addition, because there were too many secondary metabolites (Supplementary Data Table S7, Supplementary Data Fig. S5), the screening itself was subjective, which made it difficult to fully reflect the objective facts.

However, it is worth noting that this method had good stability and reliability in repetition and results analysis, despite a certain subjectivity in the screening of 30 metabolites. Similar to previous phylogenetic studies on *Chrysanthemum* and its related groups based on internal transcribed spacers (ITSs), chloroplast DNA genes [12–14], and single-copy nuclear genes, the cluster tree of 30 secondary metabolites indicated that a large number of *Chrysanthemum* and *Ajania* members were chimeric and located downstream of the cluster (Fig. 4). The coalescent species tree inferred from six nuclear gene sequences by Shen *et al*. [9] showed that *C. rhombifolium* and *C. crassum* were gathered on a very close small branch, and our results (Fig. 4) were consistent with this. Therefore, although it is difficult to provide strong evidence for phylogenetic research from our metabonomic results, they still have reasonable reference value. Furthermore, our cluster showed a somewhat regular geographic distribution pattern, which was similar to previous metabonomic studies on several *Ajania* members [51]; that is, species in similar geographic environments had closer chemotaxonomic relationships (Fig. 5).

It is worth emphasizing that our metabolomics results have multiple correlations and degrees of correlation with the results of traits and biogeography (Figs 4 and 5). We argue that this combination of metabonomics with biogeography research can well explain the special distribution pattern of some *Chrysanthemum* species and the environmental factors driving evolution, so as to explore the potential evolution and migration history more comprehensively. Expanding the number of samples and increasing biological duplication, especially for the same species from different regions, could improve the reliability and persuasiveness of the data. In addition, combined with transcriptomics and genomics, our metabolomics methods will provide more diverse and powerful evidence for phylogenetic and evolutionary research.

### Chrysanthemum glacial radiation hypothesis

Hybridization and allopolyploidy are considered important mechanisms for the origin of chrysanthemum. *C. indicum*, *C. zawadskii*, and *C. nankingense* are the direct ancestors of most chrysanthemum cultivars based on low-copy *LFY* gene sequences [52]. The phylogeny of cultivars and wild species of the genus *Chrysanthemum* using chloroplast genomes and the nuclear *LEAFY* gene suggested that geographic and ecological factors may determine the opportunities for wild species to be involved in the origin of the cultivars [4]. The biogeographic results of this study demonstrated that China, Japan, and the Korean Peninsula were the only countries and regions in the world with crossed distributions of two different color groups (Fig. 1). These crossed distribution areas eliminate geographical isolation and provide the necessary geographical conditions for wild *Chrysanthemum* species to form new species or cultivars, and the two groups in China have the largest crossed geographic area.

It is worth noting that clade 4 was special (Fig. 4, yellow branch), as the only branch composed of *Chrysanthemum* species. However, the distribution of the two species was distant; one was located in China, and the other was located in Japan. In addition, the distribution ranges of both were very narrow. *C. rhombifolium* was only found in Wushan County, Chongqing Province, while *C. crassum* was only found in Amami great island and its surrounding islands. It should be emphasized that *C. rhombifolium* was the only known species with trisegmented leaves in China, even in the Eurasian continent. The rest of the known species were distributed only in Japan and, according to distance, *C. crassum* was at the closest straight-line distance from *C. rhombifolium* among all known species of *Chrysanthemum* with trisegmented leaves in Japan. It was speculated that their common ancestors experienced long-distance migration during their evolution, and most likely migrated along the Pacific glacial track or similar tracks before forming different species and discontinuous distribution patterns.

We propose the *Chrysanthemum* Glacial Radiation Hypothesis (CGRH): *Chrysanthemum* took the potential node of panbiogeography in Central China (Fig. 2) as the radiation center, radiated around it during the glacial period, and spread and developed to East Asian islands and North America (Fig. 1) with land bridges formed by the decline in sea level during the glacial period. In the interglacial period, the distribution range shrank and formed a discontinuous distribution, and rapid speciation occurred in the process of geographic isolation and climate change caused by the alternating cycles of the glacial–interglacial period. The high mountains in Central China and its surrounding areas have become a refuge [53] for *Chrysanthemum* diversity, resulting in the preservation, continuation, evolution, and development of *Chrysanthemum* species and its allies in the alternating process of glacial–interglacial cycles.

In the Quaternary, the global continental plates were connected very close to the current plate pattern. The amplitude of the sea level exceeded 100 m [54]. The average depths of the Bering Strait (with an average water depth of ~40 m), Tsushima Strait (50–90 m), and Taiwan Strait (60 m) caused the current discontinuous distribution of *Chrysanthemum* to be <100 m. Therefore, it was not necessary to reach the Glacial Maximum, which produced land or land bridges due to falling sea levels. Our field survey results and statistics showed that 51.28, 45.95, and 71.43% of *Chrysanthemum*, *Ajania*, and *Phaeostigma* were related to rocky and rocky geomorphic habitats, respectively (Supplementary Data Table S2). Moreover, these three genera were mainly distributed in open landforms, such as coast, forest margins, thicket margins, rocky slopes, deserts, riversides, and meadows. This high adaptability to rocky habitats allowed them to radiate rapidly in the dry environment during glacial periods.

Previous studies [10] have shown that the relatively widespread tetraploid form of the *C. indicum* complex expanded its range southward in the Pleistocene, whereas diploid and other polyploid members of the complex failed to expand their ranges at these times. Our results indicate that *C. rhombifolium* (30°N) and *C. crassum* (28°N) had very similar secondary metabolites, formed a relatively independent clade in the cluster, and had similar traits. It is worth emphasizing that the former is diploid and the latter is decaploid, while other species with trisegmented leaves in Japan are octaploids, hexaploids, or other polyploids [34, 50]. Whole-genome duplication, or polyploidy, followed by gene loss and diploidization, has long been recognized as an important evolutionary force, especially in plants [55]. These changes most likely allow organisms to take advantage of new ecological opportunities or cope with new environmental challenges [56, 57]. The white–purple group, which is relatively adapted to cold and low temperatures, radiated to the high latitudes in the middle and north of the Eurasian continent; the landforms in this area (now Siberia, Russia) are mostly plains and plateaus and have relatively simple vegetation communities. *Chrysanthemum* radiated rapidly in the eastern and western directions in this area.

### Potential secondary radiation center and evolution center of *Chrysanthemum* and its allies

As refugia for biodiversity, mountains contribute disproportionately to the terrestrial biodiversity of Earth. Constituting ~25% of all land areas, mountain regions are home to >85% of the world’s amphibian, bird, and mammal species, many of which are entirely restricted to mountains [58]. The high biodiversity of certain mountainous regions reflects the interplay of multiple evolutionary mechanisms: enhanced speciation rates with distinct opportunities for coexistence and persistence of lineages, shaped by long-term climatic changes interacting with topographically dynamic landscapes [59]. For *Chrysanthemum*, our results showed that nearly 92.31% of the species were distributed in mountains and surrounding areas, which accounted for ~7.60% of the total distribution regions. The landforms of the two diversity hotspots, CSNMS and JMISI, are mostly mountainous (Table 1). Moreover, the average distribution altitude (ADA) of *Chrysanthemum*, *Ajania*, and *Phaeostigma* showed significant differences (Fig. 7), and many key traits used to define the relationship between genera, such as ray florets (RF) and corolla lobes of tubular florets (CL), were significantly correlated with the ADA (Fig. 6). Therefore, these hotspots, mainly composed of mountain landforms, may be centers of the evolution of *Chrysanthemum* and its allies, playing a key role in their divergence and evolution.

The Hengduan Mountains–Qinling Mountains (HDQ) region of China is the only region with crossed distribution of three genera in the world, and provides the potential global panbiogeographic nodes of *Chrysanthemum* (Fig. 2). This region is an important global biodiversity hotspot [60], as well as the origin, cradle, and refuge of many flora [61–63]. The local patterns of species diversity in different habitats mirror the abundance of these particular habitats in evolutionary history [64]. The number of species of *Chrysanthemum*, *Ajania*, and *Phaeostigma* was 11, 19, and 7, respectively, accounting for 29.73, 51.35, and 100.00% of the total richness, and 45.45, 78.95, and 71.43% of them are regionally endemic. Previous studies have shown that the small-scale alpine topography at low latitudes and the high altitudes at the southern end of this region provided a refuge [53] for *Chrysanthemum* and its allies during the Quaternary glacial–interglacial cycles. This suggests that HDQ is a refuge and potential secondary radiation center of *Chrysanthemum*.

According to previous results, the ancestors of Anthemideae are very likely to have originated from South Central Africa [65] and to be the origin of the *Chrysanthemum* group (*Chrysanthemum* + *Ajania* + *Opisthopappus*) in Central Asia, followed by eastward migration [9]. Our biogeographic results showed that most species of *Chrysanthemum* are distributed in East Asia and a few in the northwest of North America (Figs 1 and 2), which supports the eastward migration of *Chrysanthemum*. In addition, we emphasize that these migrations experienced rapid radiation during the Quaternary glacial period. However, our results (Figs 1 and 2, Supplementary Data Fig. S1a) showed that the migration and radiation of *Chrysanthemum* are not in a single direction; notably, some species experienced multidirectional radiation. Compared with the topographic change, the climate change of Quaternary glacial–interglacial cycles was more intense and had a shorter period. We highlight that mountain landform and environmental heterogeneity play a key role in the formation, maintenance, and evolution of *Chrysanthemum* species and diversity in this climate change. However, the Quaternary has experienced several glacial–interglacial cycles [66, 67], and we were unable to determine the precise time that the genus *Chrysanthemum* originated and began to radiate and migrate. This will require further in-depth research and evidence to explore the evolutionary history of *Chrysanthemum*.

## Materials and methods

### Statistics and identification of herbaria and documentation

Over 6000 herbaria of *Chrysanthemum*, *Ajania*, and *Phaeostigma* were identified from the National Plant Specimen Resource Center of China, Chinese Virtual Herbarium (CVH), Royal Botanic Garden Kew Herbarium Catalogue (K), Harvard University Herbaria & Libraries (HUH), Moscow State University Digital Herbarium (MW), and other herbaria worldwide. Furthermore, according to the *Flora of China* [29], *Flora URSS* [30], *Flora Europaea* [31], *Flora of Japan* [32] (Supplementary Data Table S1), and other documents [including but not limited to 33–39, 68–73], a two-way identification and comparison between the records and herbaria were carried out. According to the results, the main taxa of the three genera (Supplementary Data Tables S2 and S3) in the world were sorted and counted from the traditional view of geographic speciation.

### Field investigations and sampling

To ensure the accuracy of geographical information and descriptions of the samples, we conducted a large number of field investigations and sampling of *Chrysanthemum*, *Ajania*, and *Phaeostigma* species in different areas of China from 2016 to 2020. The sampling range included 14 provinces, autonomous regions, and municipalities, including Yunnan, Tibet, Xinjiang, Heilongjiang, and Inner Mongolia. The longitude and latitude ranged from 81.03°E to 129.44°E and 25.17°N to N47.77°N, respectively. The geographic information, habitat, and morphological characteristics of each species were investigated and recorded. The living specimens and related vouchers collected in this study were deposited in the China Chrysanthemum Germplasm Conservation Center of Nanjing Agricultural University.

### Biogeography analysis based on a geographic information system

A total of 963 global coordinate points were selected according to geographic information records of field investigations, herbaria, and documentations for biogeography analysis. Among them, 482, 399, and 82 were collected from *Chrysanthemum*, *Ajania*, and *Phaeostigma*, respectively (Supplementary Data Fig. S2). The specific geographical distribution of each species is shown in Supplementary Data Fig. S1 and the filter rules are shown in Supplementary Data Table S4. To better understand the global distribution and migration history of *Chrysanthemum*, the samples were divided into the yellow ray florets group (*C. indicum* group) and the white–purple group (*C. zawadskii* group) [13], according to the generally separated distribution of the two groups. Combined with geological events, the panbiogeography analysis was carried out based on the analysis method adopted by Moreira-Muñoz and Muñoz-Schick [40].

To explore the global diversity and potential secondary origin center of *Chrysanthemum* and its allies, the global diversity hotspots of *Chrysanthemum* and three genera (*Chrysanthemum* + *Ajania* + *Phaeostigma*) were analyzed according to the species richness and distribution range. The diversity hotspot areas were determined according to the methods of Huang *et al*. [41]. Three main rules were followed: (i) the species richness in the region exceeded 20% of the total number of species; (ii) the regions with high species richness ratios and low area ratios were selected to determine the diversity hotspot areas; and (iii) based on rules (i) and (ii), the number of endemic species was considered. The similarity of species in different diversity hotspots was measured using the Sørenson index (SI) using the following formula:

$$ \begin{align*} SI = 2c/ (a+b) \end{align*}$$

where *a* and *b* are the number of species in different hotspots, and *c* is the number of common species.

Furthermore, based on the results of digital elevation model (DEM) mapping and statistics, the elevation, geomorphology, and species distribution of diversity hotspots were analyzed, and the potential factors of diversity formation are discussed.

### Correlation analysis based on traits and environmental factors

Based on the differences in the characteristics of the 80 species (Supplementary Data Table S2; except *Chrysanthemum chalchingolicum*, *Ajania abolinii*, and *Chrysanthemum bizarre*) from the main taxa of *Chrysanthemum*, *Ajania*, and *Phaeostigma*, 31 main traits [e.g. presence/absence of ray florets (RF), degree of lignification (LD), involucral surface (IS), and corolla colors of tubular floret (CC)] were classified and coded (Supplementary Data Table S5), including 13 reproductive-related traits and 18 non-reproductive-related traits. A principal component analysis (PCA) of 31 traits [42] was performed to understand the evolutionary relationships among the three genera.

Based on previous statistics on geographic information data, the three environmental factors of altitude (ADA), latitude (ADL), and climate type (CTL) of the distribution area were assigned and coded (Supplementary Data Table S6). The altitude and latitude of the distribution were determined by the arithmetic mean value of the upper and lower extreme values of each species, and the climate type was mainly determined by the mean annual precipitation [43, 44], which can be queried in the main global distribution area of each species as the main quantitative index. We then conducted a correlation analysis based on trait coding and environmental factor coding.

### Non-targeted metabolomics research based on GC–MS

A total of 38 species of *Chrysanthemum* (19 species), *Ajania* (15 species), and *Phaeostigma* (4 species) which mainly came from China and Japan were selected for metabolomics research (Supplementary Data Table S7). All species were preserved in the China Chrysanthemum Germplasm Conservation Center of Nanjing Agricultural University and grown under the same natural conditions. In the same vegetative growth period, three mature leaves in the middle of the stems were randomly selected on a sunny day (9:00–11:00). After picking, they were quickly stored in liquid nitrogen for enzyme quenching, and then stored at −80°C until use.

Plant leaves were frozen in liquid nitrogen and ground into a powder. Ethyl acetate was added (1 mL per 0.2 g tissue), and nonyl acetate was added (0.003% w/v) as an internal standard to obtain an organic mixture [45]. Extraction was performed at 25°C with continuous shaking at 300 rpm and 25°C for 4 hours. Then, 100–200 μl of extract was placed in a brown injection bottle (Agilent, USA) for GC–MS separation and identification of compounds. This procedure was repeated in triplicate for each species.

The compounds, mainly composed of terpenoids, were analyzed using an Agilent 7000D Triple Quadrupole GC/MS. Separation was performed on an HP-5 capillary column (30 m × 0.25 mm i.d. × 0.25 μm thickness) under the following conditions: high-purity helium (He2, 99.99%) was used as the carrier gas for gas chromatography, the carrier gas flow rate was 1 ml min^−1^, split injection was used, split ratio was 5:1, and split flow rate was 5 ml min^−1^. The quenching gas (2.25 ml min^−1^) and back-blowing gas (3 ml min^−1^) were also helium, and the flow rates were 2.25 and 3 ml min^−1^, respectively; the collision gas was nitrogen with a flow rate of 1.5 ml min^−1^ and the injection port temperature was set to 260°C, which was the best temperature obtained by the orthogonal test. The injection volume of the extract was 1 μl. Qualitative analysis of the compounds was performed using the Agilent MassHunter qualitative analysis workflow BO8.00 and Agilent MassHunter Qualitative Analysis Navigator BO8.00. Quantification was performed as previously described [46, 47].

To elucidate the evolutionary relationship of the three genera from the identified metabolites, the following screening rules and analysis methods were adopted: (i) priority was given to the chromatographic peaks with a qualitative matching score ≥90 and accurate identification; (ii) on the basis of rule (i), the top 10 chromatographic peaks with peak area from large to small of each species were selected for quantitative analysis; (iii) on the basis of meeting rule (ii), the top 30 metabolites with high frequency and high diversity in all the tested samples (38 species, a total of 114 samples) were selected for quantitative analysis. The analysis mainly included PCA, orthogonal partial least-squares discriminant analysis (OPLS-DA), and cluster analysis based on the ratio of the peak area of target metabolites to the peak area of the internal standard.

### Statistical analysis

For the distribution altitude of each species (including subspecies, varieties, and forms) (Supplementary Data Table S2), the maximum, minimum, and average values of the distribution altitude recorded in documentation or field investigations were used as the statistical data set. When there was no specific elevation in the documentation, the value was estimated according to the geographic range of distribution. If the value could not be estimated, statistical analysis was not carried out. Due to the lack of relevant records in some areas, extreme values and the complete range of distribution latitude were regarded as the latitude distribution range in the latitude analysis. Origin 2019 (OriginLab, USA) and SPSS 25.0 (IBM, USA) were used for statistical analysis of the data set. Because the number of species in the three genera was different and did not conform to the normal distribution, an independent samples non-parametric test (Kruskal–Wallis test and Jonckheere–Terpstra test) was used for significance analysis (*P* < .05). ArcGIS 10.5 (Esri USA) and the GCS-WGS-1984 coordinate system were used for vector mapping. Google’s global satellite vector layer and DEM using Shuttle Radar Topography Mission 3 (SRTM 3, NASA and NIMA, USA) were used for mapping analysis. PCA was performed using Origin 2019 (OriginLab, USA), while correlation analysis and cluster analysis were performed using R 3.6.1 (R Core Team, 2016). OPLS-DA was performed with SIMCA 14.1 (Umetrics Inc., Kinnelon, NJ, USA) software for 200 permutation fittings.

## Acknowledgements

This work was financially supported grants from National Natural Science Foundation of China (31730081, 32001354, 31872149), The National Key Research and Development Program of China (2019YFD1001500, 2018YFD1000401), the China Postdoctoral Science Foundation (2019 M661871), the earmarked fund for Jiangsu Agricultural Industry Technology System, and a project funded by the Priority Academic Program Development of Jiangsu Higher Education Institutions.

## Author contributions

X.C. planned and designed the research, conducted the fieldwork, sampling and data collection, carried out analyses, and wrote the initial draft. Y.J. and W.Z. participated in GC–MS tests. H.W., J.J., and F.C. revised manuscript. F.C. planned the research.

## Data availability

The data underlying this article are available in the article and in its online supplementary material.

## Conflict of interest

The authors declare that they do not have any possible conflicts of interest.

## Supplementary data

Supplementary data is available at *Horticulture Research* online.

## Supplementary Material

Web_Material_uhac153
