# Supplementary material for: Biogeographic and metabolic studies support a glacial radiation hypothesis during Chrysanthemum evolution
Source: Hortic Res. 2022 Jul 6;9:uhac153. doi: 10.1093/hr/uhac153 (PMC9527600; doi:10.1093/hr/uhac153)
Supplement: Web_Material_uhac153 [file web_material_uhac153.docx]

**Supplementary Information**

**Table S1.** List of abbreviations for references and herbarium.

| Abbreviations | Reference/Herbarium |
| --- | --- |
| FE | Flora Europaea |
| FC | Flora of China |
| FJ | Flora of Japan |
| FU | Flora URSS |
| FNA | Flora of North America |
| CVH | National Plant Specimen Resource Center of China |
| E | Royal Botanic Garden Edinburgh Herbarium Catalogue |
| HUH | Harvard University Herbaria & Libraries |
| K | Royal Botanic Garden Kew Herbarium Catalogue |
| LE | Botanicheskii Institut im.V.L.Komarova,Saint Petersburg |
| TAI | Taiwan University herbarium museums |
| TI | Herbarium of the University of Tokyo |

**Table S2.** Taxa and distribution of *Chrysanthemum*, *Ajania*, and *Phaeostigma*.

| № | Taxa | Distributions | Habitats & Altitudes | Reference & Vouchers |
| --- | --- | --- | --- | --- |
|  | ***Chrysanhemum*** |  |  |  |
| 1 z | *C. arcticum* L. |  |  |  |
|  | subsp. *arcticum* | Ru(Ar,E1,S5,S6,S7), Ja, Us(AR,NW), Ca(AR,NW) | Stony tundra and seashores, stony and sandy places, gravel beds, grassy patches along coasts | FE Vol. 4 P169; FNA Vol. 19 P534-536; FU Vol. 26 P386-387; MW(0159133); HUH(00589512) |
|  | subsp. *polare* (Hultén) Tzvelev. | Ru(Ar,S5,S6,S7) | Similar to that of *C. arcticum* subsp. *arcticum*, near the Arctic | FE Vol. 4 P169; FNA Vol. 19 P534-536;MW(0143751); MW(0143762) |
|  | subsp. *Yezoens* (Maek.) H. Ohashi & Yonek. | Ja | Rocks of seashores | Ohashi & Yonekura, 2004; FJ P1184 |
| 2*z | *C. argyrophyllum* Y. Ling | Ch(Shanxi,Henan) | On rocks of mountain slopes, mountain tops (1400-2100 m) | FC;CVH(PE00533027,PE00533023); Chen&Wang0005 |
| 3 i | *C. arisanense* HayataIcon.Pl. Formosan. | Ch(Taiwan) | Slightly shady roadside slopes, on exposed roadside (1600-1860 m) | FC;CVH(PE00533030, PE01913076,PE 01106978); TAI(KYO_00022268); E(00016835) |
| 4**i | *C. aromaticum*  (Q.H.Liu et S.F.Zhang)  J.Zhou et J.Y.Chen | Ch  (Hubei:Shennongjia) | Slopes in high altitude (2100-2800 m) | Chen, 2012; CVH(CCAU0011483); Chen00107 |
| 5 z | *C. bizarre*  C.Z. Shen | Ch  (Hunan:Zhangjiajie) | Rocky cliffs, rocky mountain slopes (800-1480 m) | Meng et al., 2020 |
| 6 z | *C. chalchingolicum* Grubov. | Mo |  | Botanicheskii Zhurnal. Moscow & Leningrad (St. Petersburg) 1972;  Bremmer & Humphries, 1993 |
| 7*z | *C. chanetii*  H. Léveillé | Ch,Ru,Mo,KP | Grasslands, forest margins on mountain slopes, floodlands, by ditches (300-2700 m) | FC; CVH(abundant); K(000891717); K(000891708(-10)) |
| 8**z | *C. crassum*  (Kitam.) Kitam. | Ja |  | Ohashi & Yonekura,2004; Kitamura,1967; Nakata et al., 1987; Chen0607 |
| 9**i | *C. dichroum*  (C. Shih.) H. Ohashi & Yonekura | Ch(Hebei:Neiqiu) | Mountain slopes | FC; CVH(PE02015839); Chen0003 |
| 10*i | *C. foliaceum*  (Peng., Shih.et Zhang.)  Wang. et Hou. | Ch(Shandong:Jinan) | Rocky slope forests margins (100-300 m) | Shih et al.,1999; FC; CVH(PE00935970) Chen&Wang0013 |

continued Table S2

| 11*i | *C. glabriusculum* (W.W.Smith.)Ha.-Ma. | Ch(Yunnan,Sichuan, Shanxi) | Mountain slopes (940-2600(3300) m) | FC;CVH(PE00533349,  PE00533342); Chenxi0026 |
| --- | --- | --- | --- | --- |
| 12 z | *C. horaimontanum* Masamune. | Ch(Taiwan) | Rocky slopes (1200-1400 m) | FC; TAI(119187) |
| 13 i | *C. hypargyreum*  Diels. | Ch(Shanxi,Sichuan) | Meadows on mountain slopes (1400-3850 m) | FC; CVH(PE00569068(-69)) |
| 14**i | *C. indicum*  L. |  |  |  |
| ** | var. *indicum* | Ch,Ru,In,Ja,KP,My | Grasslands on mountain slopes, thickets, wet places by rivers, fields, roadsides, saline places by seashores, under shrubs (100-2900(3200) m) | Ohashi & Yonekura, 2004; Kitamura,1967; Nakata et al., 987; FC; CVH(abundant), K(001118855,001118859); Zhao02002,Zhao.LS04-01; Chenxi0037,Chenxi0020 |
|  | var. *coreanum* H.Léveillé. | Ch,KP |  | Chen, 2012; E(00417217(-19)) |
| ** | var. *acutum* Kitamura. | Ch(Shandong:Taishan) |  | Zhao, 2007; Li. TS04-01 |
|  | var. *albescen* Makino. | Ja |  | Ohashi & Yonekura, 2004 |
|  | var. *huludaoense* G.Y. Zhang | Ch  (Liaoning:Huludao) |  | Chen, 2012; FC |
|  | var. *iyoens* | Ja | Hillsides, stony landforms (250-305 m) | Ohashi & Yonekura, 2004;Kitamura,1967;  Nakata et al.,1987 |
| ** | var.*maruyamanum* Kitam. | Ja |  | Ohashi & Yonekura, 2004; Kitamura, 1967;  Nakata et al., 1987; Chen0105035, Chen0105036 |
|  | var.*tsurugisanense* K. | Ja | Mountaintops, on limestones (1800 m) | Kitamura, 1967; Ohashi & Yonekura, 2004 |
| 15**z | C. *japonense*  Nakai. |  |  |  |
| ** | var. *japonens* | Ja | Seashores | Ohashi & Yonekura, 2004; Kitamura,1967; Nakata et al., 1987; Chen0605, Chen0105017 |
| ** | var. *ashizuriense* Kitam. | Ja |  | Ohashi & Yonekura, 2004; Kitamura,1967; Chen0105037，Chen0105038 |

continued Table S2

| 16*i | *C. lavandulifolium* (Fischer ex Trautvetter) Makino. |  |  |  |
| --- | --- | --- | --- | --- |
| * | var. *lavandulifolium* | Ch,KP | Mountain slopes, rocks, river valleys, riverbanks,wastelands, hilly lands (600-2800 m) | FC; CVH(HBNU10017072, HBNU10017074);E(00557781(-88)); Chenxi0025 |
|  | var. *discoideum* (Hand.-Mazz.)Shih. | Ch(Sichuan) | Mountain slopes | FC |
| * | var. *tomentellum* (Hand.-Mazz.) Ling et Shih | Ch(Yunnan) | Slope forests margins (2600-3100 m) | FC; CVH(PE00533977(-87)); Chenxi0033 |
| 17 i | *C. longibracteatum* (C.Shih. G.F.Peng & S.Y.Jin) J.M.Wang et Y.T.Hou | Ch(Shandong) | Woodland margins, along paths (100 m) | Shih et al.,1999; FC;  CVH (PE00935971(-75)) |
| 18**z | *C. makinoi*  Matsum. & Nakai. | Ja | Hills, moutains, on limestones | Ohashi & Yonekura, 2004; Kitamura,1967; Nakata et al.,1987; FJ:P1185-1186； K(000891702); Chen0602 |
| 19*z | *C. maximowiczii* (Komarov.) Tzvel. | Ch,Ru(S6),KP | Mountain slopes, nearby lakes, sand dunes (900-1500 m) | FC;CVH(PE00533989(-95)), MW(0143729(-34)); Chenxi0036 |
| 20*z | *C. mongolicum*  Y. Ling. | Ch,Ru,Mo | Rocky mountain slopes, cliffs (1500-2500 m) | FC; CVH(PE00533996(-4001); Chen&Wang0012 |
| 21 z | *C. morii*  Hayata. | Ch(Taiwan) | Limestone cliffs (rare) (400-2400 m) | FC |
| 22*z | *C. naktongense*  Nakai. | Ch,Ru(S6),KP | Grasslands, forests margins, stony forests ((200)1400-1700 m) | FC;CVH(abundant),  MW(0143669(-73)); Chenxi0024, Chen&Wang0020 |
| 23**i | *C. nankingense* Hand.-Mazz. | Ch(Nanjing) | Hilly forest margins, mountain slopes (40-400 m) | Chen, 2012; Ren et al, 2014; Song et al., 2018; Chen0001 |
| 24**z | *C. okiense*  Kitam. | Ja |  | Ohashi & Yonekura, 2004；Chen0603 |
| 25*z | *C. oreastrum*  Hance | Ch,Ru(S6),KP | Meadows, gravel meadows (1800-3000 m) | FC; CVH(PE02035825, PE01552239);MW(0143665(-67),0075542(-43));  Chen&Wang0019 |
| 26**z | *C. ornatum* Hemsl. | Ja |  |  |
| ** | var. *ornatum* | Ja | Seashores | Ohashi & Yonekura, 2004; Kitamura, 1967;  Nakata et al., 1987; FJ:P1184; K(000891698); Chen0105031(-033) |

continued Table S2

|  | var. *tokarense* (M.Hotta & Y.Hirai)H.Ohashi & Yonek. | Ja |  | Ohashi & Yonekura, 2004 |
| --- | --- | --- | --- | --- |
| 27 z | *C. parvifolium* C.C. Chang | Ch(Guizhou) | Rocky slopes, near small rivers | FC |
| 28 i | *C. potentilloides* Handel-Mazzetti | Ch(Shaanxi Shanxi) | Foothills of low mountains (1000-1500 m) | FC;CVH(PE00030004,PE00030006); HUH(GH00004809) |
| 29*z | *C. rhombifolium* (Y.Ling. & C. Shih.) H.Ohashi & Yonekura | Ch(Chongqing(Wushan)) | Mountain slopes, Stony slope shrubs margins (800-1200 m) | FC; Chenxi0035 |
| 30*i | *C.seticuspe* (Maxim.)Hand.-Mazz | Ch,Ja,KP | Shade or moist ecotype | Ohashi & Yonekura, 2004; Hirakawa, et al., 2019; FC; CVH(abundant) |
|  | f. *seticusp* | Ja |  | Ohashi & Yonekura, 2004 |
| ** | f. *boreale*  (Makino) H.Ohashi & Yonek. | Ch,Ja,KP |  | Ohashi & Yonekura, 2004; FJ:1187;  Chen0601 |
| 31**z | *C. vestitum*  (Hemsley) Stapf. |  |  |  |
| ** | var. *vestitum* | Ch(Anhui,Henan, Hubei,Shaanxi) | Low mountain slopes, hills (340-1500 m) | FC; CVH(NAS00486822(-26)); Chen0006 |
| * | var. *latifolium* J.Zhou & Jun Y.Chen | Ch(Anhui,Henan) | Shaded slopes, streamsides | FC; Chen00061 |
| 32 z | *C. wakasaense*  Shimot.ex Kitam. | Ja | Seashores, mountain slopes | Ohashi & Yonekura, 2004; FJ:1185-1186; Chen0105030 |
| 33**z | *C. weyrichii*  (Maxim.) Miyabe & T. Miyake. | Ja,Ru(S7) | Rocks of seashores, rocky slopes, rocks | Ohashi & Yonekura, 2004; FJ:1184, FU Vol. 26 P383 K(000891715) |
|  | var.littorale (Maekawa) Kudo. | Ja, Ru(S7) | Rocks of seashores, rocky slopes, rocks | FU Vol. 26 P384 |
| 34 z | *C. xeromorphum*  Khokhr. | Ru(S5,S7) |  | Khokhriakov, 1977; MW(0143632(-34),0161765) |
| 35 i | *C. yantaiense* M.Sun et J.T.Chen | Ch(Shandong:Yantai) | Barren reefs and rock crevices and infertile and thin soils (4-15 m) | Chen et al., 2018 |
| 36*z | *C. yezoense* Maek. | Ja | Rocks of seashores | Ohashi & Yonekura, 2004;  FJ: 1184 |
| 37 z | *C. yoshinaganthum* Makino ex Kitam. | Ja | Streamsides | Ohashi & Yonekura, 2004, FJ:1185 |

continued Table S2

| 38**z | *C. zawadskii*  Herbich. | Ch,Ru(E1,E10,E6,S1,S2,S3,S4,S5,S6), Mo,KP,Ja,EU(Cz,Po) | Grassland, inter forest grasslands, under forest, stream edges, stony slopes (850-1800 m) | FC;FE Vol. 4 P169; FU Vol. 26 P376-377; Ohashi & Yonekura, 2004;CVH(abundant);MW(abundant);K(000891699(-701)); HUH(GH00589644);Chen0007, Chen000701(-02) |
| --- | --- | --- | --- | --- |
| 39 z | *C. zhuozishanense*  L.Q. Zhao & J.Yang | Ch(Inner Monlolia) | Wet northern slopes of Zhuozishan (Albasi) in Ordos (2000-2140 m) | Zhao et al, 2014 |
|  | ***Ajania*** |  |  |  |
| 1 | *A. abolinii*  Kovalevsk. | Central Asia |  | Ohashi & Yonekura, 2004 |
| 2* | *A. achilleoides* (Turczaninow) Poljak. ex Grubov. | Ch,Mo | Desertification stony slopes, desert steppes, steppes | FC; CVH(abundant), MW(abundant), Chen&Wang0014 |
| 3* | *A. adenantha*  (Diels) Ling. et Shin. | Ch(Yunnan:Lijiang) | Stony slope shrubs margins, mountain slopes, stony alpine meadows (3000-3700 m) | FC; CVH(abundant); E(abundant); Chenxi0001 |
| 4 | *A. alabasica*  H.C.Fu | Ch(Inner Mongolia) | Rocky slopes | FC |
| 5 | *A. amphisericea* (Handel-Mazzetti) C.Shih | Ch(Sichuan:Kangding,Tianquan) | Mountain slopes (1700-2300 m) | Shih, 1994; FC |
| 6 | *A. brachyantha*  Shih | Ch(Xizang(Tibet):Nyêmo) | Mountain slopes (3500-3600 m) | FC; CVH(abundant) |
| 7** | *A. breviloba*  (Franch.ex Hand-Mazz.) Ling. et Shih | Ch(Yunnan) | Open places in forests, gravelly places on mountain slopes (2800-4100 m) | FC; CVH(abundant), HUH(abundant);  Xie. LJ 06-01 |
| 8 | *A. elegantula* (W.W.Smith.) Shih | Ch(Yunnan) | Open stony pasture, on ledges  of cliffs (3000 m) | FC; K(000891734); E(00413540) |
| 9* | *A. fastigiata*  (C.Winkl.) Poljak. | Ch(Xinjiang), Af,Ka,Ky,Ta | Steppes, semideserts, forest understories (900-2260 m) | FC; CVH(abundant); MW(abundant); Chen&Wang0015 |
| 10* | *A. fruticulosa*  (Ledeb.) Poljak. | Ch,Ka,Ru(S2),Mo,Tu | Deserts, desert steppes (500-4400 m) | FC; CVH(abundant); MW(abundant); HUH(00124016);  Chenxi0030 |
| 11 | *A. gracilis*  (J.D.Hooker & Thomson) Poljak. | Ch(Xizang(Tibet)), Ky,Ta | Stony slopes (above 3000 m) | FC; FU Vol. 26 P407; CVH(PE00386991(-93),HNWP41112(,16));K(000891775, 000891719(-21)) |
| 12 | *A. grubovii*  Muldashev | Mo |  | Ohashi & Yonekura, 2004; Bremer K & Humphries, 1993; MW(0192082(-86)) |

continued Table S2

| 13 | *A. hypoleuca*  Y.Ling. ex C. Shih | Ch(Gansu,Sichuan) | Roadsides (600-700 m) | Shih, 1994; FC; CVH(PE00386994) |
| --- | --- | --- | --- | --- |
| 14* | *A. khartensis*  (Dunn) C.Shih in C.Shih & G.X.Fu | Ch,In(N) | Alpine gravel meadows, mountain slopes (2500-5300 m) | FC; CVH(abundant); HUH(abundant);TI(Registerd No.20104173, 10007069); Chenxi0007,Chen&Wang0004 |
| 15** | *A. kinokuniense* (Shimot. & Kitam.) X.Chen | Ja | Seashores | Ohashi & Yonekura, 2004; FJ:1185 |
| 16 | *A. korovinii*  Kovalevs. | Ka |  | Ohashi & Yonekura, 2004；MW(0883978(-79)) |
| 17 | *A. latifolia*  C.Shih | Ch(Sichuan) | Mountain slopes  (3100 m) | FC; PE(00387057(-58)) |
| 18* | *A. myriantha*  (Franchet) Y.Ling. ex C.Shih in C.Shih & G.X.Fu | Ch,Bh | Stony or rocky slope, forests margins, mountain slopes, river valleys  (2200-3600 m) | FC; CVH(abundant); HUH(abundant); Chenxi0006, Chenxi0014, Chenxi0010 |
| 19* | *A. nematoloba* (Handel-Mazzetti) Y.Ling & C.Shih | Ch(Gansu,Qinghai) | Slope shrubs, mountain slopes (1700-2300 m) | FC; CVH(abundant); HUH(00124025(-26)); Chen&Wang0007 |
| 20 | *A. nitida*  C.Shih | Ch(Sichuan) | Dry mountain slopes  (3900 m) | FC; CVH(PE00420028, KUN0036885) |
| 21 | *A. nubigena*  (Wallich ex Candolle) C.Shih in C.Shih & G.X.Fu | Ch(Xizang(Tibet), Sichuan,Yunnan,Gansu), Bh,In,Ne | Mountain slopes  (3900-4100 m) | FC; CVH(PE00420029(-30), PE01519085); K(000891722(-24)); TI(abundant) |
| 22** | *A. pacifica* (Nakai) K.Bremer & Humphries | Ja | Seashores | Bremer & Humphries, 1993; Ohashi & Yonekura,2004; FJ:1185; Nakta et al., 1987; K(000891732); Zhao.0401, Chen0104001(-08) |
| 23* | *A. pallasiana*  (Fischer ex Besser) Poljak. | Ch(Heilongjiang,Jilin), Ru(S6),KP,Ja | Stony forests margins, rocky ledges of cliffs thickets, mountain slopes, on rocks of high mountains (200-2900 m) | Ohashi & Yonekura, 2004; FC; CVH(abundant), K(000891731); MW(abundant);HUH(00274118(-19)); Chen&Wang0021 |
| 24* | *A. parviflora*  (Grün.) Ling | Ch(Beijing, Hebei, Shanxi,Inner Mongolia) | Rocky slope shrubs margins, low mountains, hills (1400 m) | FC; CVH(abundant),  Chenxi0023 |
| 25** | *A. potaninii*  (Krasch.) Poljak. | Ch(Sichuan,Gansu,Shaanxi) | Mountain slopes, forests, river valleys, hills (950-3500 m) | FC; CVH(abundant); HUH(abundant);Zhao. SC 06-06 |
| 26* | *A. przewalskii*  Poljak. | Ch(Gansu,Sichuan, Qinghai,Ningxia) | Shrubs and forest margins, grassland, on rocks (2800-4500 m) | FC; CVH(abundant); Chen&Wang0011 |

continued Table S2

| 27* | *A. remotipinna* (Handel-Mazzetti) Y.Ling. & C.Shih | Ch(Shaanxi,Gansu,  Sichuan,Xizang(Tibet)) | Rocky slope Thickets margins, mountain slopes  (2200-3800 m) | FC; CVH(abundant); Chenxi0021 |
| --- | --- | --- | --- | --- |
| 28 | *A. rupestre*  (Matsum. & Koidz.) X.Chen | Ja | On rocks of high mountains (1200-2600 m) | Ohashi & Yonekura, 2004; FJ:1185 |
| 29* | *A. scharnhorstii*  (Regel &Schmalhausen) Tzvelev. | Ch(Gansu,Qinghai, Xinjiang,Xizang(Tibet))，Ru,Ky,Ta,Ka | Fissures of rocks on mountain  slopes, calcareous talus slopes, thickets (3900-5100 m) | FC; FU Vol. 26 P409; CVH(abundant); MW(0883980(-81)) |
| 30 | *A. semnanensis*  Sonboli | Iran(Semnan) | Rocky and rubbly slopes of mountains (1500-2800 m) | Sonboli et al., 2013 |
| 31* | *A. sericea*  C. Shih | Ch(Yunnan:Dali) | Stony forests margins (2700-3000 m) | FC; HUH(00124052);  Chenxi0003 |
| 32* | *A. shiwogiku*  (Kitam.) X.Chen | Ja | Seashores | Ohashi & Yonekura, 2004; Kitamura,1967; Nakata et al., 1987; FJ:P1184-1185; Chen0104010 |
| 33* | *A. tenuifolia* (Jacquemont ex Candolle) Tzvelev. | Ch(Qinghai,Gansu, Xizang(Tibet),Sichuan),In(NW),Ne | Grasslands on mountain slopes (2200-4600 m) | FC; CVH(abundant); MW(0749572,0755379); K(000250066);TI(Registered No.9962944); Chen&Wang0008 |
| 34* | *A. trilobata*  Poljak. | Ch(Xingjiang) | Fissures of rocks by rivers  (3200 m) | FC; CVH(abundant); Chen&Wang0016 |
| 35 | *A. trifida*  (Turcz.) Muldashev | Ch(InnerMongolia),Mo | Desert grasslands (900-1400m) | FU; FC; CVH((abundant);  MW(0192087(-92)) |
| 36 | *A. tripinnatisecta*  Y. Ling & C. Shih | Ch(Sichuan) | Mountain slopes  (3200-3300 m) | FC; CVH(abundant); MW(0749570) |
| 37 | *A. truncata* (Handel-Mazzetti)  Y.Ling ex C.Shih | Ch(Sichuan) | Streamsides, wastelands, slopes (1900-2100 m) | 石铸, 1994; FC; PE(00420279(-83)) HUH(00004812(-13)) |
|  | ***Phaeostigma*** |  |  |  |
| 1 | *P. junnanicum*  (Poljak.) X.Chen | Ch(Yunnan N) | 3200 m | FC; Pellicer et al.*,* 2009; LE |
| 2* | *P. purpureum*  (C.Shih.) G.Y.Rao. et Y.Huang | Ch(Xizang(Xizang(Tibet))) | Alpine meadows, thickets, gravel mounds (4800-5300 m) | FC; Huang et al, 2017; CVH(abundant), Chen&Wang0001 |
| 3* | *P. quercifolium* (W.W.Smith.) Muldashev. | Ch(Sichuan,Yunnan) | Forest understories, thickets (3200-3900 m) | FC; Huang et al, 2017; Muldashev,1981;CVH(abundant), HUH(00124070); Chenxi0002, Chenxi0005 |
| 4* | *P. ramosum* (C.C.Chang) G.Y.Rao et Y.Huang | Ch(Sichuan,Xizang(Tibet)) | Stony slope forests margins, mountain slopes, river valleys (2900-4600 m) | FC; Huang et al, 2017; CVH(abundant),HUH(00292526(,28),00311989), Chenxi0018 |

continued Table S2

| 5* | *P. salicifolium*  (Mattfeld ex Rehder & Kobuski.) Muldashev. | Ch(Gansu,Shaaxi,Sichuan,Qinghai,Yunnan) | Stony slope forests margins, Mountain slopes  (2600-4600m) | FC; Muldashev, 1981; CVH(abundant),HUH(00123110,00236704);Chen&Wang0010 |
| --- | --- | --- | --- | --- |
| 6 | *P. tibeticum*  (J.D.Hooker &Thomson ex C.B.Clarke) G.Y.Rao  et Y.Huang | Ch(Xizang(Tibet),Sichuan),In,Ka,Ta,Pa, Ky | Mountain slopes  (3900-4700 m) | FC; Huang et al, 2017; CVH(abundant),MW(abandant) |
| 7* | *P. variifolium* (C.C.Chang)Muldashev. | Ch(Shaanxi,Hubei,Sichuan) | Alpine screes, rocky slopes (1500-3500 m) | FC; Muldashev, 1981; CVH(abundant),HUH(00274365(-66),00274130);Chenxi0022 |

Note: The number angle sign * indicates that the living plant specimens was obtained by this research personally, and the number angle sign ** indicates that the living plant specimens was obtained by the supervisor and previous research of our research group; the sign z means *C. zawadskii* group, and i means *C. indicum* group; for the abbreviations of countries and regions in this table, please refer to Table S1 and Supporting Table S3.

**Table S3.** List of abbreviations for country/region/direction.

| Abbreviations | Country/region/direction |
| --- | --- |
| Af | Afghanistan |
| Am | America |
| Bh | Bhutan |
| Ca | Canada |
| Ch | China |
| Cz | Czechoslovakia |
| EU | Europe |
| In | India |
| Ir | Iran |
| Ja | Japan |
| Ka | Kazakhstan |
| KP | Korean Peninsula |
| Ky | Kyrgyzstan |
| Mo | Mongolia |
| My | Myanmar |
| Ne | Nepal |
| Pa | Pakistan |
| Po | Poland |
| Ta | Tajikistan |
| Tu | Turkmenistan |
| Ru | Russia |
| Ru(E1) | Eastern Europe,Northern region |
| Ru(E4a) | Eastern Europe,Moscow region |
| Ru(E6) | Eastern Europe,Central forest-and-steppe region |
| Ru(E10) | Eastern Europe,Eastern region |
| Ru(S1) | Siberia,Western Siberia |
| Ru(S2) | Siberia,Altai & Sayany Mountains |
| Ru(S3) | Siberia,Central Siberia |
| Ru(S4) | Siberia,Baikal & Transbaikal region |
| Ru(S5) | Siberia,Yakutia |
| Ru(S6) | Siberia,Far East |
| Ru(S7) | Siberia,Chukotka & Kamchatka |
| N | North |
| NW | Northwest |
| W | West |
| AR | Arctic |

**Table S4.** Filter rules and priority level of global coordinate points.

| № | Filter rules | priority level |
| --- | --- | --- |
| 1 | Herbariums or records collected by this study | ***** |
| 2 | Herbariums with detailed distribution coordinates | **** |
| 3 | Records with detailed distribution coordinates | *** |
| 4 | Herbariums with detailed geographical distribution description | *** |
| 5 | Records with detailed geographical distribution description | *** |
| 6 | Herbariums with geographical distribution accurate to the country | ** |
| 7 | Records with geographical distribution accurate to the country | ** |
| 8 | Herbariums with geographical distribution accurate to the region | ** |
| 9 | Records with geographical distribution accurate to the region | ** |
| 10 | Records without confirmed herbariums | * |
| 11 | Herbariums without distribution description | * |

Note: The selection of all coordinate points was based on the correctness of the herbarium and documentation identification. Then, the distribution coordinate points are selected according to the priority (*number). For the same species, the coordinate points with very close geographical distribution and low priority in the global dimension were deleted.

**Table S5.** Traits codes of *Chrysanthemum*, *Ajania*, and *Phaeostigma.*

| № | Traits | Codes |
| --- | --- | --- |
| 1 | Lignified Degree(LD) | Herbs 0/Subshrubs 1/Shrubs 2 |
| 2 | Plant form(PF) | Creeping form 0/Cushion form or low tufted 1/Rosette 2/Erect or diffuse 3 |
| 3 | Stems(ST) | Solitary(few branched ) 0/Not solitary(branched) 1 |
| 4 | Creeping rhzizomes(CR) | None 0/Exist 1 |
| 5 | Leaf Texture(LT) | Thin-chartaceous 0/Not thin-chartaceous 1 |
| 6 | Leaf scar(LS) | None 0/Exist 1 |
| 7 | Leaf divisions(LI) | None(or shallowly dentate) 0/ 1-(subtripalmate)pinnatisect 1 / 2-(subtripalmate)pinnatisect 2/ 3-(subtripalmate)pinnatisect 3 |
| 8 | Degree of leaf divisions(DD) | Entire 0/ Margin coarsely dentate 1/ Shallowly-Cleft 2/ Half-Cleft 3/ Deeply-cleft 4/ Totally-cleft 5 |
| 9 | Primary lateral segments(PS) | 0-paired 0/1 1-paired(trifid) 1/ 2 -paired(pentalobed) 2/ Over 3-paired(include 3-paired) 3 |
| 10 | Ultimate segments(or cleft teeth) shapes(US) | None or obtusely dentate 0/ Obliquely triangular or elliptic 1/ arrowly elliptic1 or lanceolate 2/Linear or approximately linear 3 |
| 11 | Same/Similar color on both surfaces of leaf(SC) | No 0/Yes 1 |
| 12 | Adaxially leaf colors(AD) | Green 0/ Gray-white(or gray-green) 1 |
| 13 | Abaxially leaf colors(AB) | Green 0/ Gray-white(or gray-green) 1 |
| 14 | Indumentum(ID) | Glabrous(Subglabrous) 0/Sparsely pubescent(or villous) 1/ Densely pubescent(or villous) 2/Densely and thickly sericeous(or tomentos) 3 |
| 15 | Lateral expansion of petiole base(LE) | None 0/ Exist 1 |
| 16 | Density of leaves arranged on stem(DA) | Sparsely 0/Medium 1/Densely 2 |
| 17 | Leaf margin(LM) | Plane 0/ Refexed or Circinate 1 |
| 18 | Leaf margin white(LW) | No 0/Yes 1 |
| 19 | Capitula(CA) | Solitary(or few) 0/ Some,with sparsely compound-corymbose at apices of branches 1/ Numerous, with densely compound-corymbose at apices of branches 2 |
| 20 | Involucres(IV) | Campanulate 0/Cup-shaped(or dish-shaped) 1 |
| 21 | Involucral colours(IC) | Straw-colored 0/Not straw-colored 1 |
| 22 | Involucral surface(IS) | Glossy(nearly glossy) 0/Not glossy 1 |
| 23 | Phyllaries scarious margin(PM) | Narrow 0/wide 1 |
| 24 | Phyllaries margin colours(PC) | White 0/Light-brown(or yellow-brown) 1/Dark-brown 2 |
| 25 | Entire phyllaries(EP) | No 0/Yes 1 |
| 26 | Ray florets(RF) | None 0/ Exist 1 |
| 27 | Corolla lobes of tubular floret(CL) | Outward-curved 0/Erect 1 |
| 28 | Corolla colors of tubular floret(CC) | Yellow 0/Not yellow 1 |

continued Table S5

| 29 | Sessile glands of tubular floret(SG) | Large and obvious 0/ Not large and obvious 1 |
| --- | --- | --- |
| 30 | Same color on bisexual floret and marginal female floret(SF) | No 0/Yes 1 |
| 31 | Brownish style-branches(BS) | No 0/Yes 1 |

**Table S6.** The codes of environmental factors.

| № | Environmental factors | Codes |
| --- | --- | --- |
| 1 | Average distribution altitude(ADA) | 0-999.99 m 0/1000-1999.99 m 1/2000-2999.99 m 2/3000-3999.99 m 3/4000-5000 m 4 |
| 2 | Average distribution latitude(ADL) | N 20-25° 0/N 25.01-30° 1/N 30.01-35° 2/N 35.01-40° 3/N 40.01-45° 4/Over N 45° 5 |
| 3 | Climate type(CLT) | Moist (Mean annual precipitation＞800 mm）0/  Semi-moist (Mean annual precipitation 400-800 mm) 1/  Semi-arid (Mean annual precipitation 200-400 mm)2/  Arid (Mean annual precipitation＜200 mm) 3 |

**Table S7.** Taxa, number of peaks and sum of peak areas based on GC-MS of 38 species from *Chrysanthemum*, *Ajania*, and *Phaeostigma.*

| № | Taxa | Number of peaks(Mean±SD) | | Sum of peak areas (Mean±SD)×10^8^ | |
| --- | --- | --- | --- | --- | --- |
| 1 | *C.argyrophyllum* | 129.33±6.65 | | 4.25±1.11 | |
| 2 | *C.chanetii* | 97.67±2.87 | | 0.89±0.11 | |
| 3 | *C.crassum* | 182.33±10.87 | | 7.93±0.69 | |
| 4 | *C.dichroum* | 147.00±8.29 | | 7.13±1.03 | |
| 5 | *C.foliaceum* | 160.67±7.32 | | 3.87±0.39 | |
| 6 | *C.glabriusculum* | 160.67±6.60 | | 4.78±0.47 | |
| 7 | *C.indicum* | 178.33±3.86 | | 8.35±0.76 | |
| 8 | *C.japonense* | 168.33±7.41 | | 4.03±0.07 | |
| 9 | *C.lavandulifolium* | 132.67±2.62 | | 4.06±0.22 | |
| 10 | *C.maximowiczii* | 139.33±6.60 | | 3.54±0.32 | |
| 11 | *C.mongolicum* | 120.33±7.04 | | 2.07±0.40 | |
| 12 | *C.naktongense* | 125.67±14.64 | | 2.11±0.04 | |
| 13 | *C.nankingense* | 111.33±7.41 | | 1.69±0.50 | |
| 14 | *C.okiense* | 150.00±8.83 | | 8.24±1.80 | |
| 15 | *C.oreastrum* | 93.00±3.38 | | 0.77±0.08 | |
| 16 | *C.ornatum* | 152.33±8.73 | | 4.43±0.76 | |
| 17 | *C.rhombifolium* | 152.00±5.89 | | 7.70±0.91 | |
| 18 | *C.vestitum* | 173.00±8.04 | | 4.90±0.50 | |
| 19 | *C.zawadskii* | 112.33±2.05 | | 1.86±0.16 | |
| 20 | *A.achilleoides* | 212.67±11.15 | | 15.78±2.69 | |
| 21 | *A.adenantha* | 183.67±0.47 | | 6.31±1.59 | |
| 22 | *A.fastigiata* | 182.67±10.21 | | 9.26±1.62 | |
| 23 | *A.fruticulosa* | 169.67±7.59 | | 9.70±1.56 | |
| 24 | *A.khartensis* | 175.33±5.19 | | 9.07±0.81 | |
| 25 | *A.myriantha* | 183.67±7.59 | | 18.33±1.48 | |
| 26 | *A.nematoloba* | 130.00±3.56 | | 3.72±0.45 | |
| 27 | *A.pacifica* | 201.33±13.22 | | 10.37±2.52 | |
| 28 | *A.pallasiana* | 163.33±2.05 | | 5.37±0.34 | |
| 29 | *A.parviflora* | 197.33±2.06 | | 7.33±1.08 | |
| 30 | *A.potaninii* | 198.33±6.18 | | 10.46±0.99 | |
| 31 | *A.przewalskii* | 182.67±4.71 | | 17.54±1.80 | |
| 32 | *A.remotipinna* | 204.33±5.44 | | 20.10±1.40 | |
| 33 | *A.sericea* | 151.00±11.34 | | 5.20±1.03 | |
| 34 | *A.tenuifolia* | 180.00±2.16 | | 19.46±1.84 | |
| 35 | *P.quercifolium* | 128.33±12.97 | 4.75±0.45 | |  |
| 36 | *P.ramosum* | 162.67±9.18 | 7.48±1.58 | |  |
| 37 | *P.salicifolium* | 157.33±5.56 | 5.35±1.16 | |  |
| 38 | *P.variifolium* | 168.00±7.12 | 11.12±1.65 | |  |

**Table S8.** 30 secondary metabolites of *Chrysanthemum*, *Ajania*, and *Phaeostigma*.

| No. | Secondary metabolites | Molecular  formula | CAS |
| --- | --- | --- | --- |
| 1 | Benzene, 1-methyl-3-(1-methylethyl)- | C10H14 | 535-77-3 |
| 2 | 3-Carene | C10H16 | 13466-78-9 |
| 3 | .beta.-Phellandrene | C10H16 | 555-10-2 |
| 4 | Bicyclo[3.1.0]hexane, 4-methylene-1-(1-methylethyl)- | C10H16 | 3387-41-5 |
| 5 | Bicyclo[3.1.1]heptane, 6,6-dimethyl-2-methylene-, (1S)- | C10H16 | 18172-67-3 |
| 6 | Camphene | C10H16 | 79-92-5 |
| 7 | Cyclohexene, 4-methylene-1-(1-methylethyl)- | C10H16 | 99-84-3 |
| 8 | D-Limonene | C10H16 | 5989-27-5 |
| 9 | (+)-2-Bornanone | C10H16O | 464-49-3 |
| 10 | Bicyclo[3.1.0]hexan-3-one, 4-methyl-1-(1-methylethyl)-, [1S-(1.alpha.,4.beta.,5.alpha.)]- | C10H16O | 471-15-8 |
| 11 | 5-Isopropyl-2-methylbicyclo[3.1.0]hexan-2-ol | C10H18O | 546-79-2 |
| 12 | Bicyclo[2.2.1]heptan-2-ol, 1,7,7-trimethyl-, (1S-endo)- | C10H18O | 464-45-9 |
| 13 | endo-Borneol | C10H18O | 507-70-0 |
| 14 | Eucalyptol | C10H18O | 470-82-6 |
| 15 | L-.alpha.-Terpineol | C10H18O | 10482-56-1 |
| 16 | Bicyclo[3.1.1]hept-2-en-6-ol, 2,7,7-trimethyl-, acetate, [1S-(1.alpha.,5.alpha.,6.beta.)]- | C12H18O2 | 50764-55-1 |
| 17 | 2,4-Di-tert-butylphenol | C14H22O | 96-76-4 |
| 18 | Phenol, 3,5-bis(1,1-dimethylethyl)- | C14H22O | 1138-52-9 |
| 19 | Ambrosin | C15H18O3 | 509-93-3 |
| 20 | (+)-epi-Bicyclosesquiphellandrene | C15H24 | 54274-73-6 |
| 21 | (1R,2S,6S,7S,8S)-8-Isopropyl-1-methyl-3-methylenetricyclo[4.4.0.02,7]decane-rel- | C15H24 | 18252-44-3 |
| 22 | Bicyclo[5.2.0]nonane, 2-methylene-4,8,8-trimethyl-4-vinyl- | C15H24 | 242794-76-9 |
| 23 | Copaene | C15H24 | 3856-25-5 |
| 24 | Cyclohexane, 1-ethenyl-1-methyl-2,4-bis(1-methylethenyl)-, [1S-(1.alpha.,2.beta.,4.beta.)]- | C15H24 | 515-13-9 |
| 25 | .gamma.-Muurolene | C15H24 | 30021-74-0 |
| 26 | Naphthalene, 1,2,3,4,4a,5,6,8a-octahydro-7- methyl-4-methylene-1-(1-methylethyl)-, (1.alpha.,4a.beta.,8a.alpha.)- | C15H24 | 39029-41-9 |
| 27 | Tricyclo[4.4.0.0(2,7)]decane, 1-methyl-3-methylene-8-(1-methylethyl)-, stereoisomer | C15H24 | 20479-06-5 |
| 28 | (3S,3aR,3bR,4S,7R,7aR)-4-Isopropyl-3,7-dimethyloctahydro-1H-cyclopenta[1,3]cyclopropa[1,2]benzen-3-ol | C15H26O | 23445-02-5 |
| 29 | Selin-6-en-4.alpha.-ol | C15H26O | 118173-08-3 |
| 30 | (1R,4aR,7R,8aR)-7-(2-Hydroxypropan-2-yl)-1,4a-dimethyldecahydronaphthalen-1-ol | C15H28O2 | 4666-84-6 |


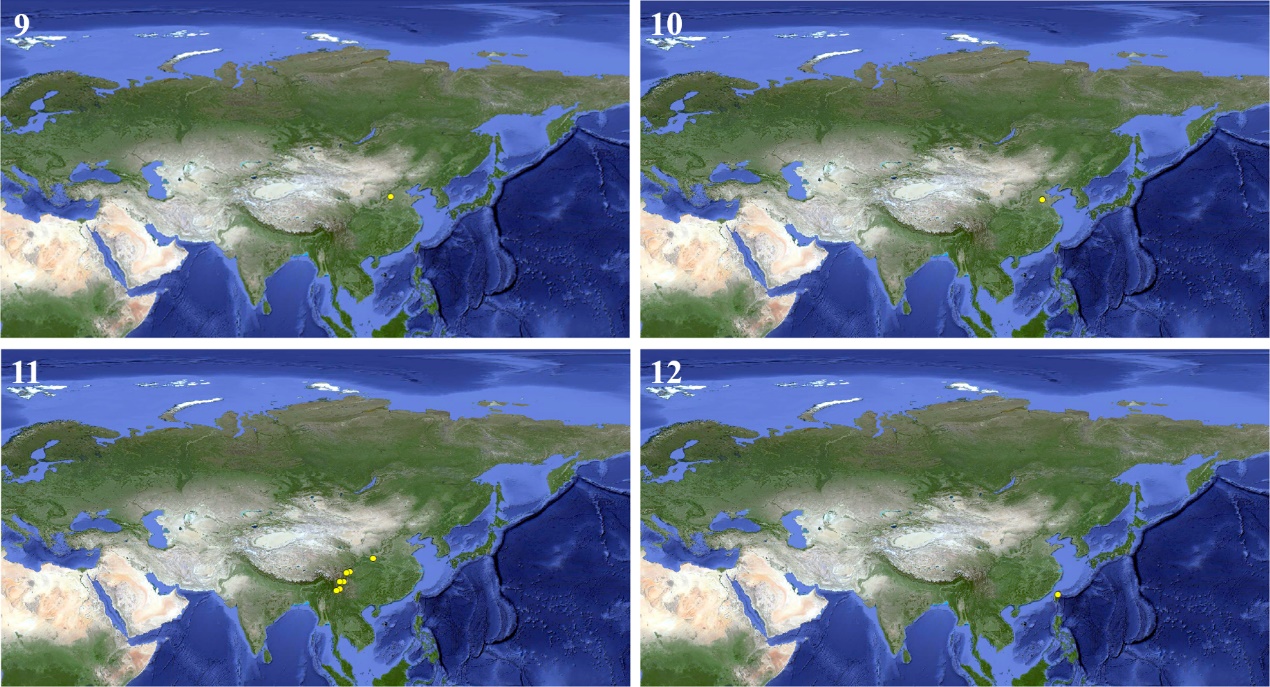

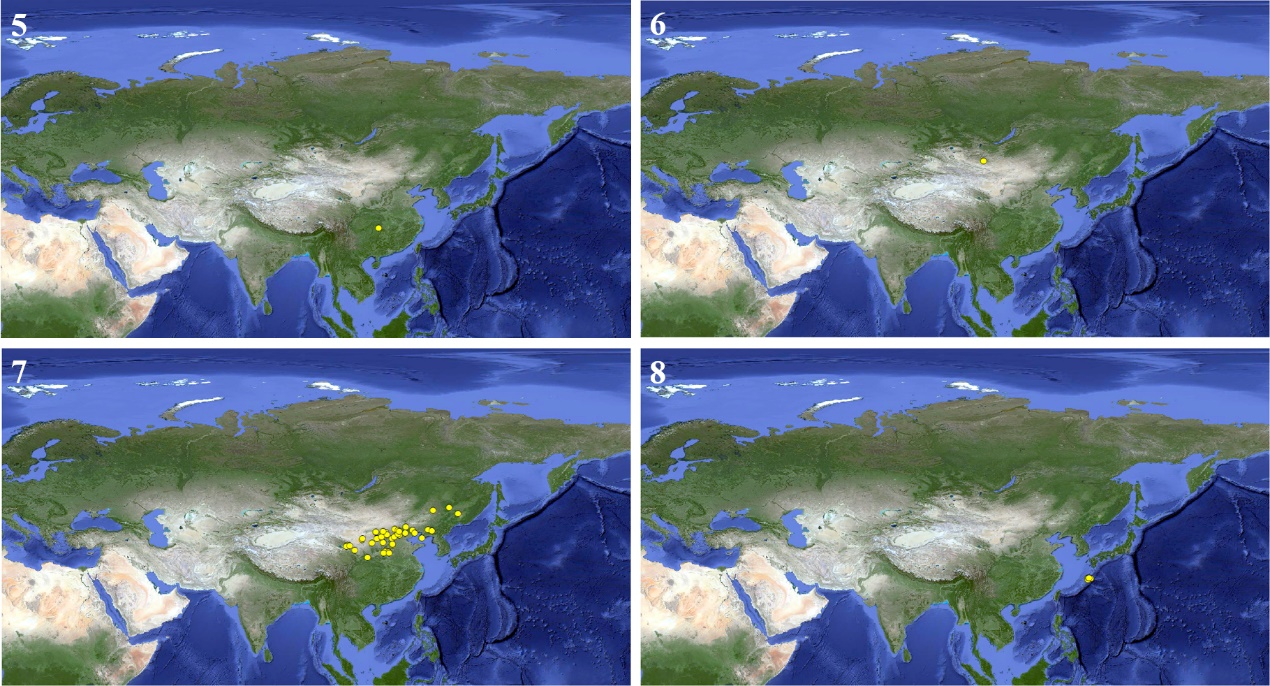

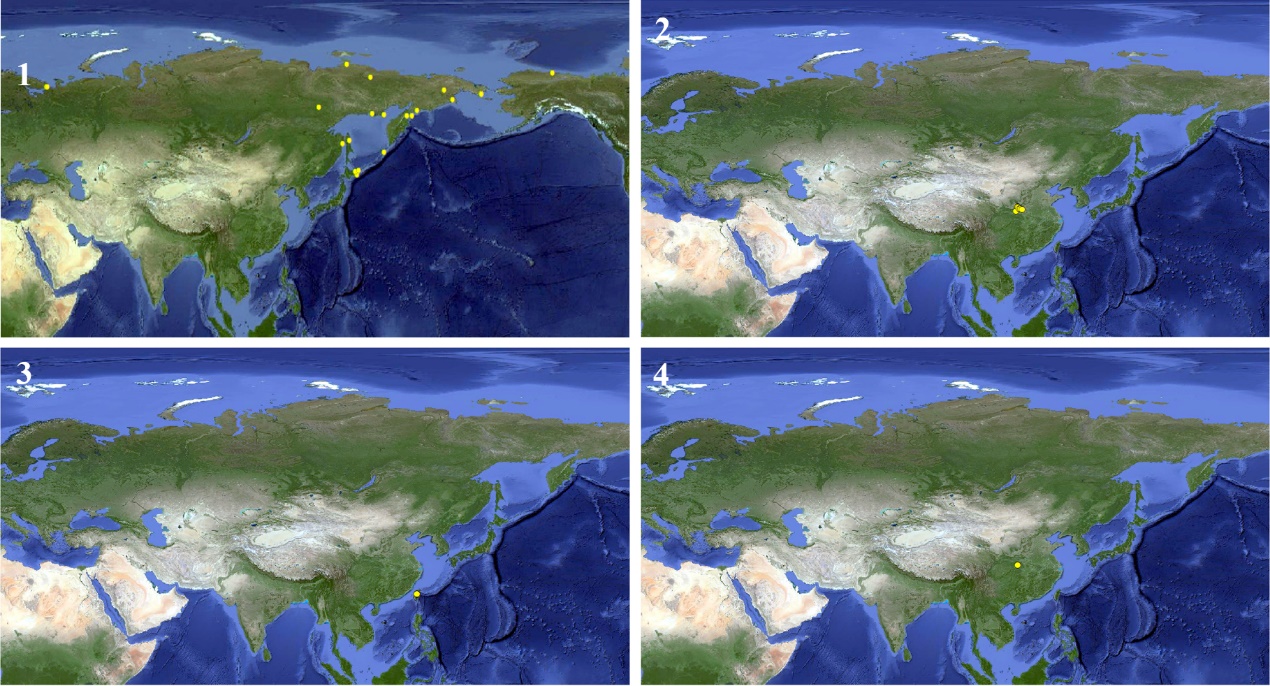


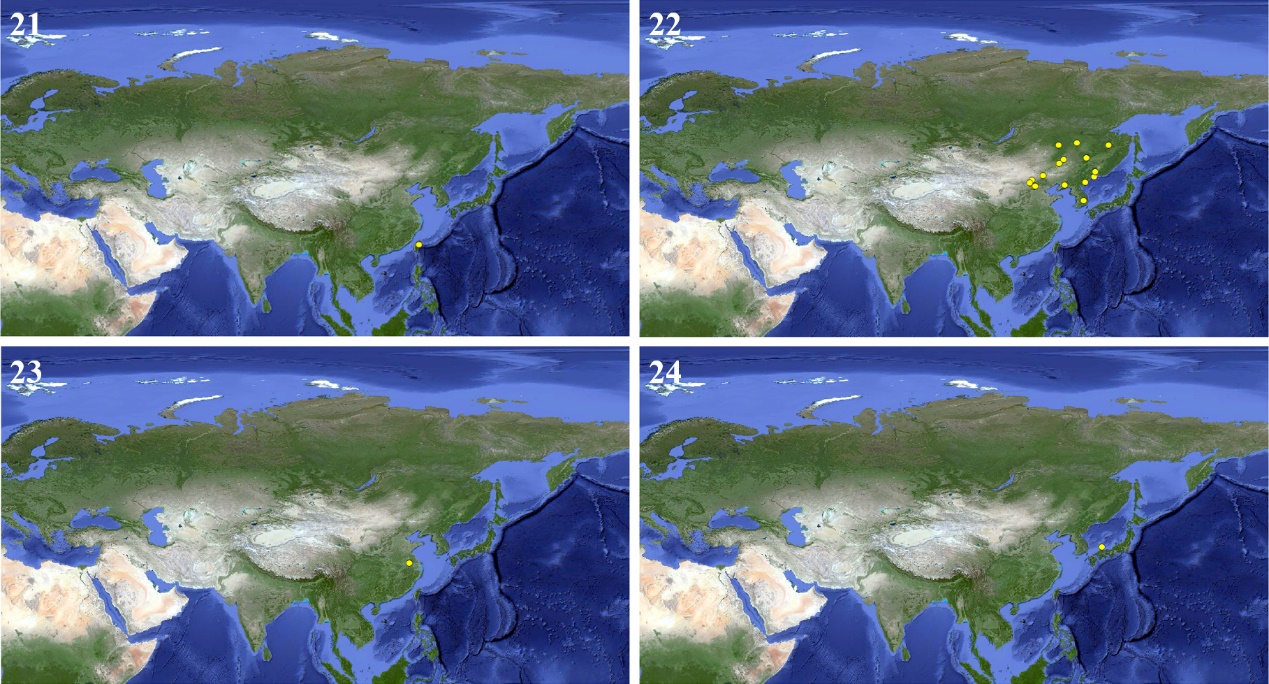

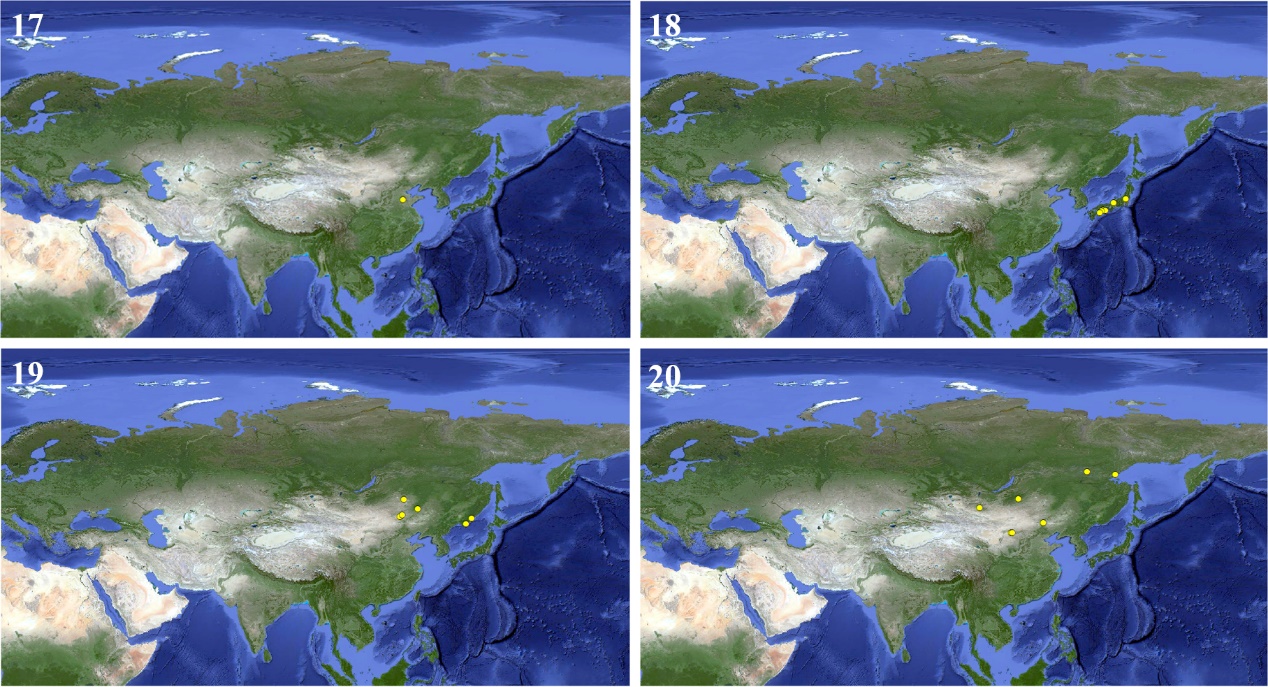

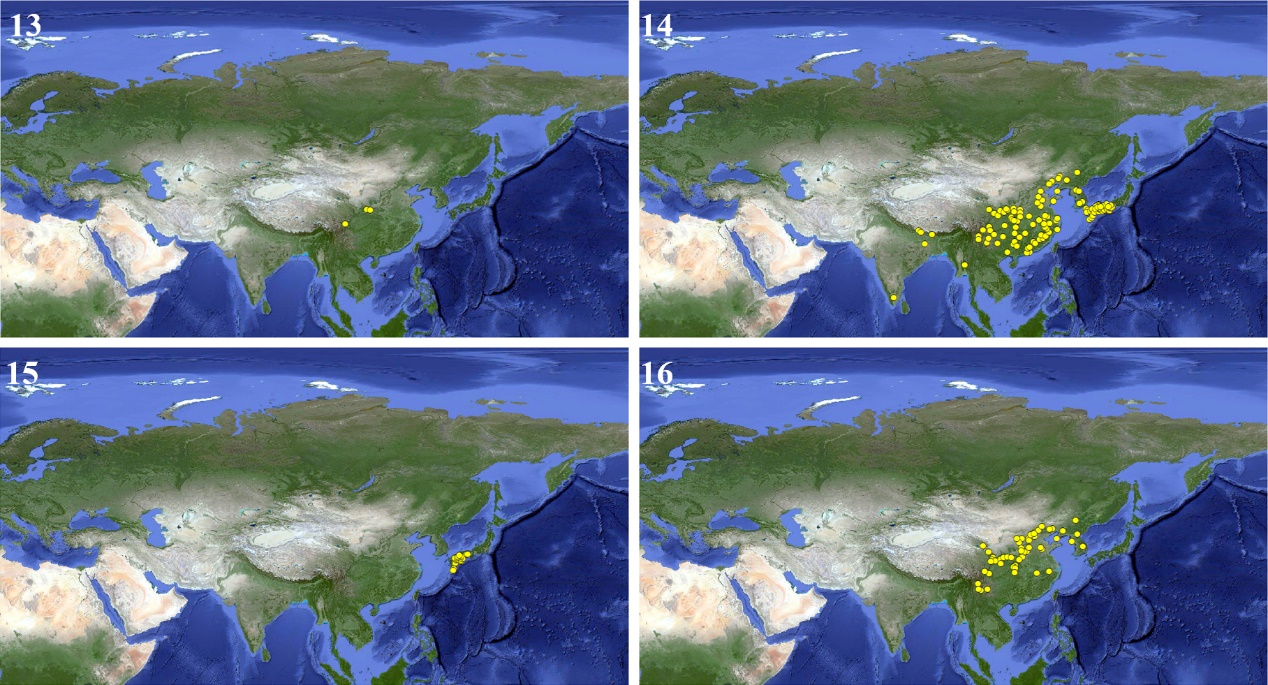


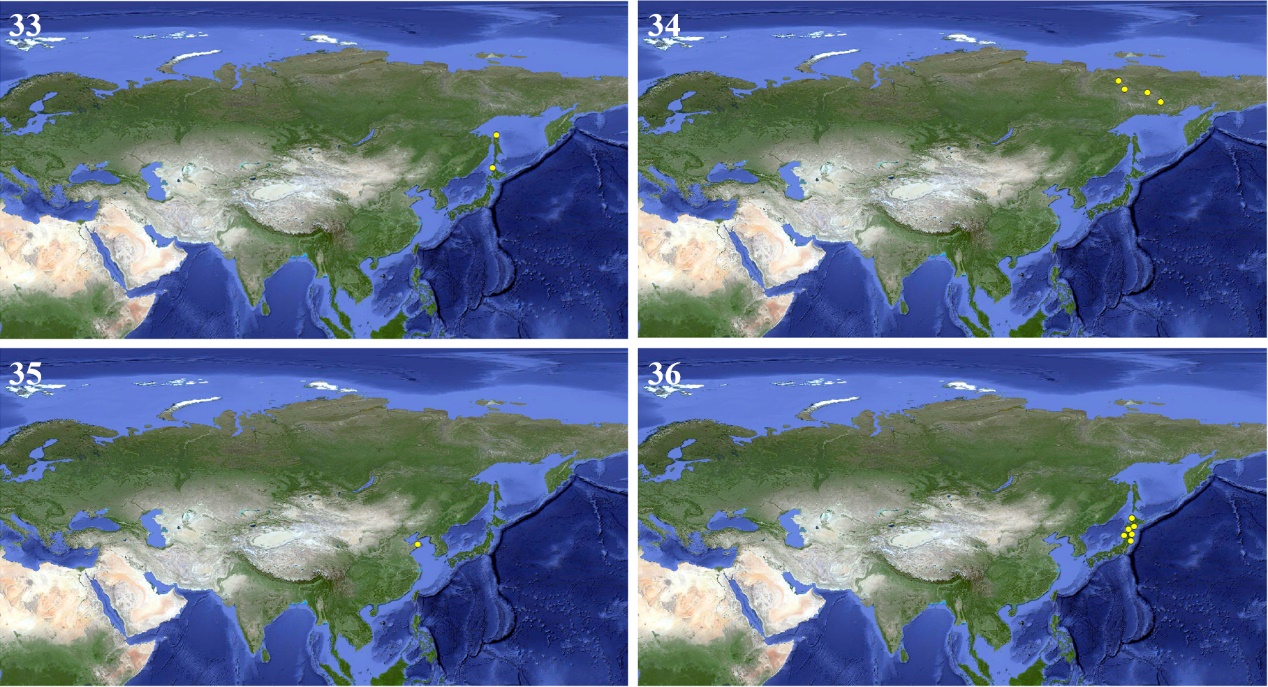

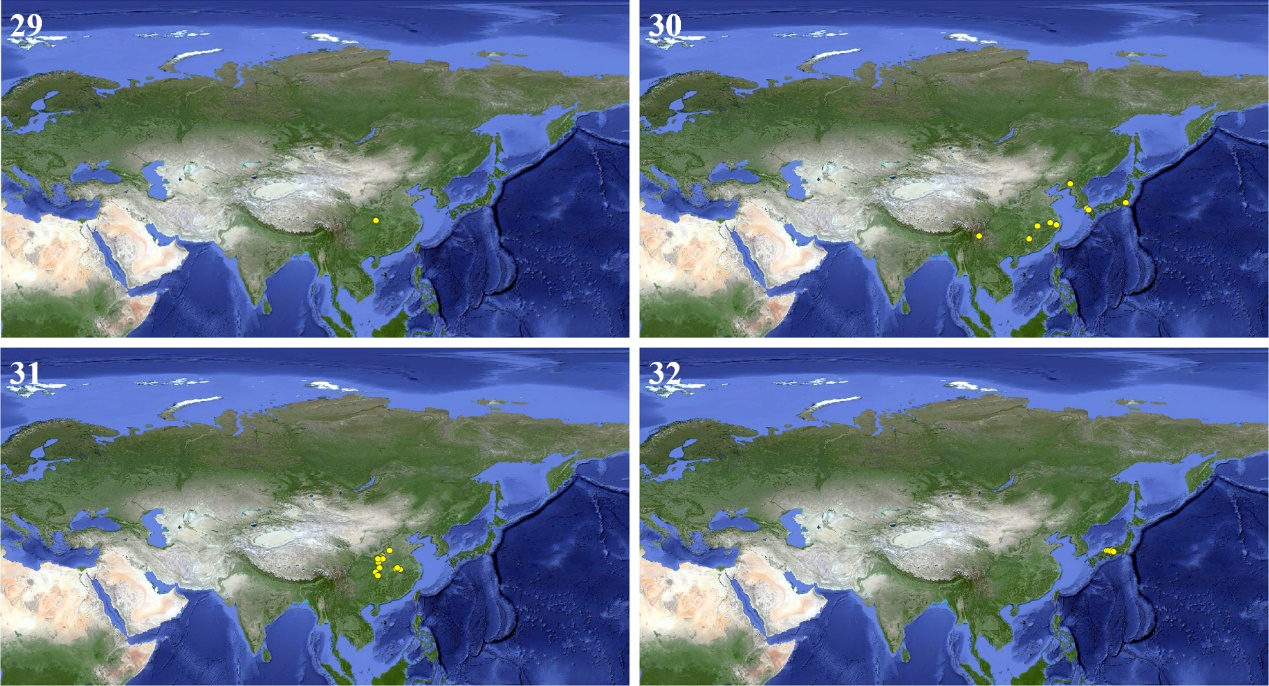

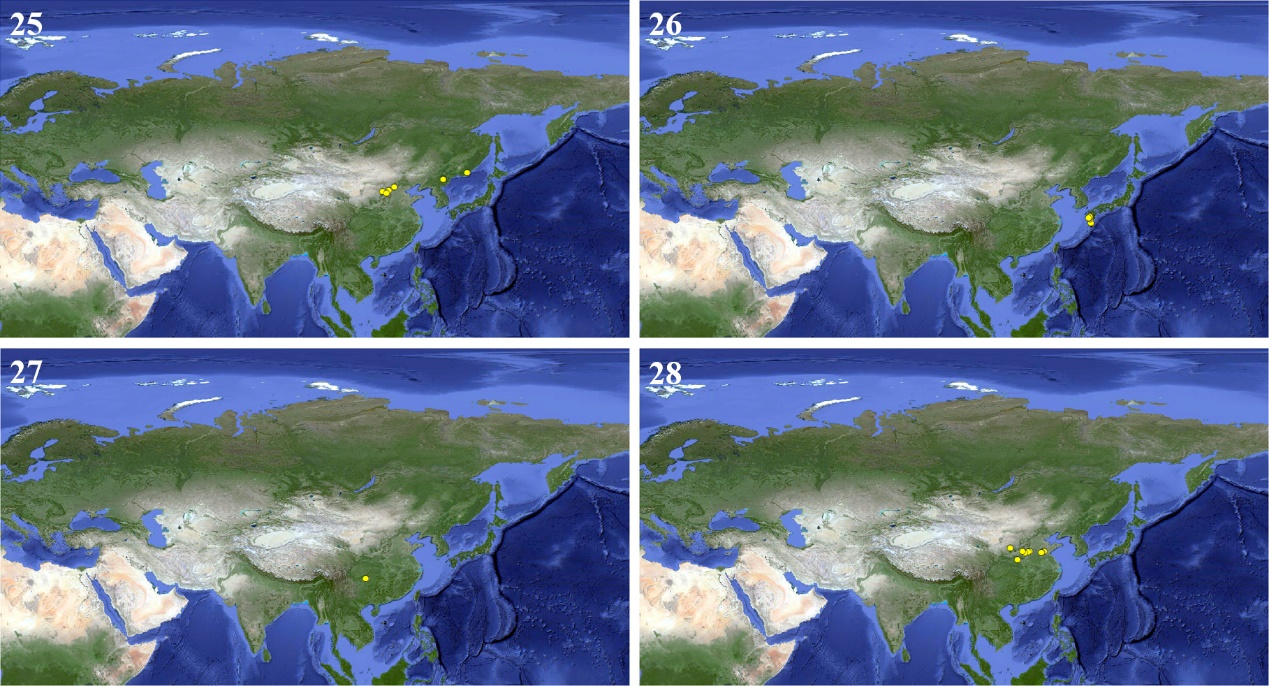


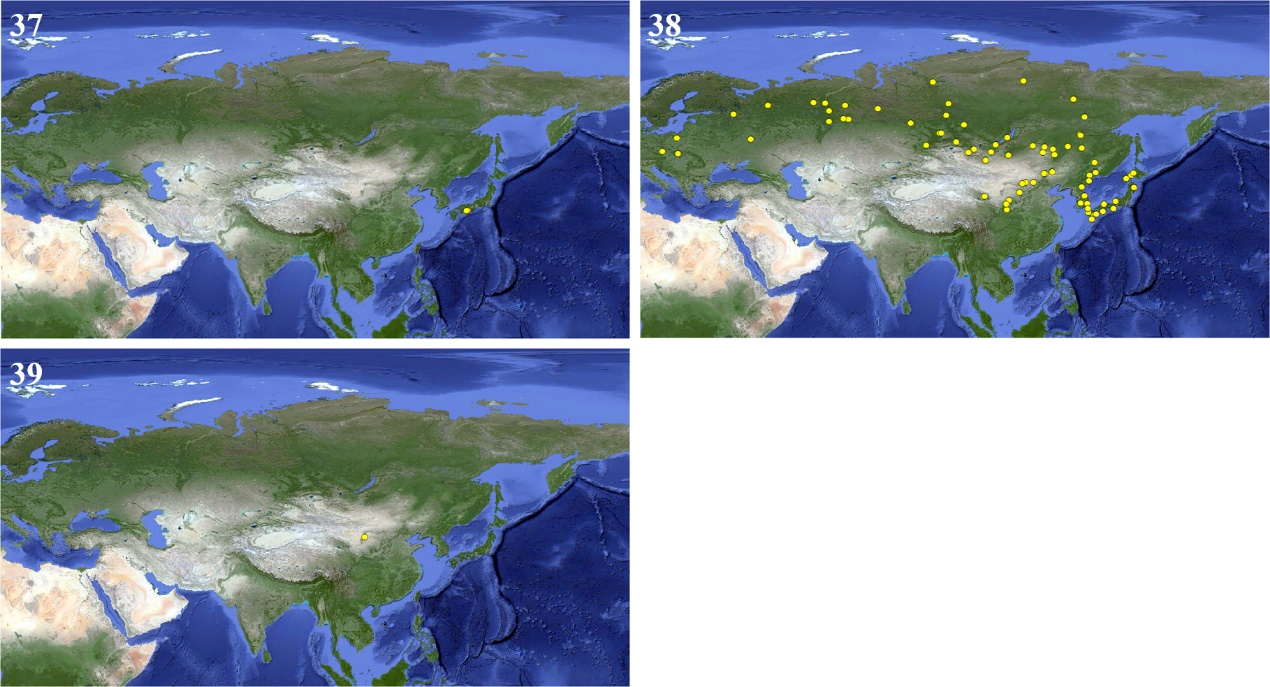


**Fig. S1** (a) Distributions of each species of *Chrysanthemum*, the species numbers corresponded to Table S2


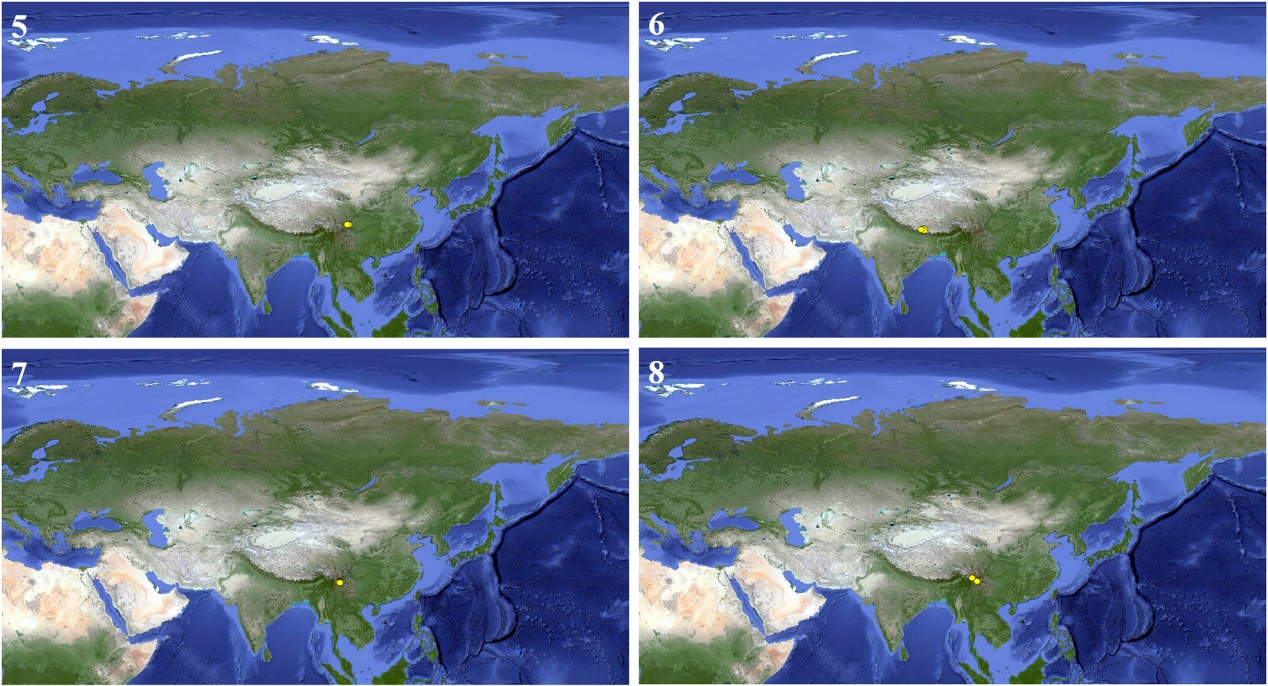

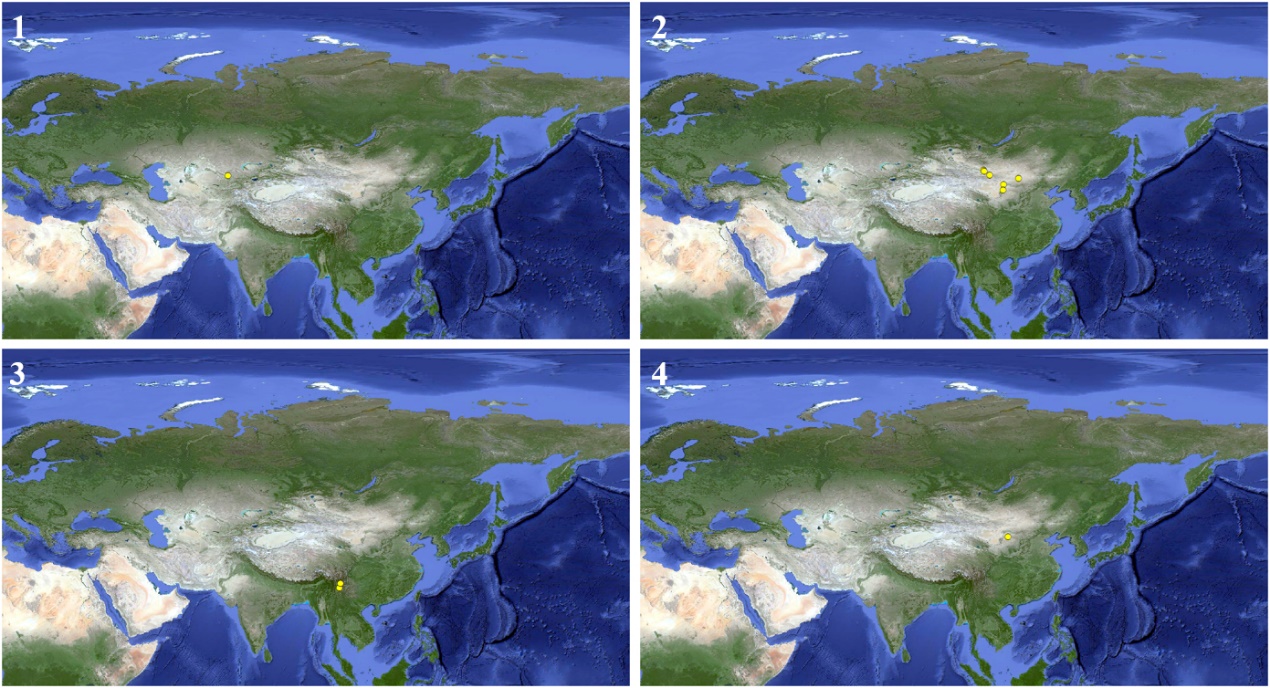


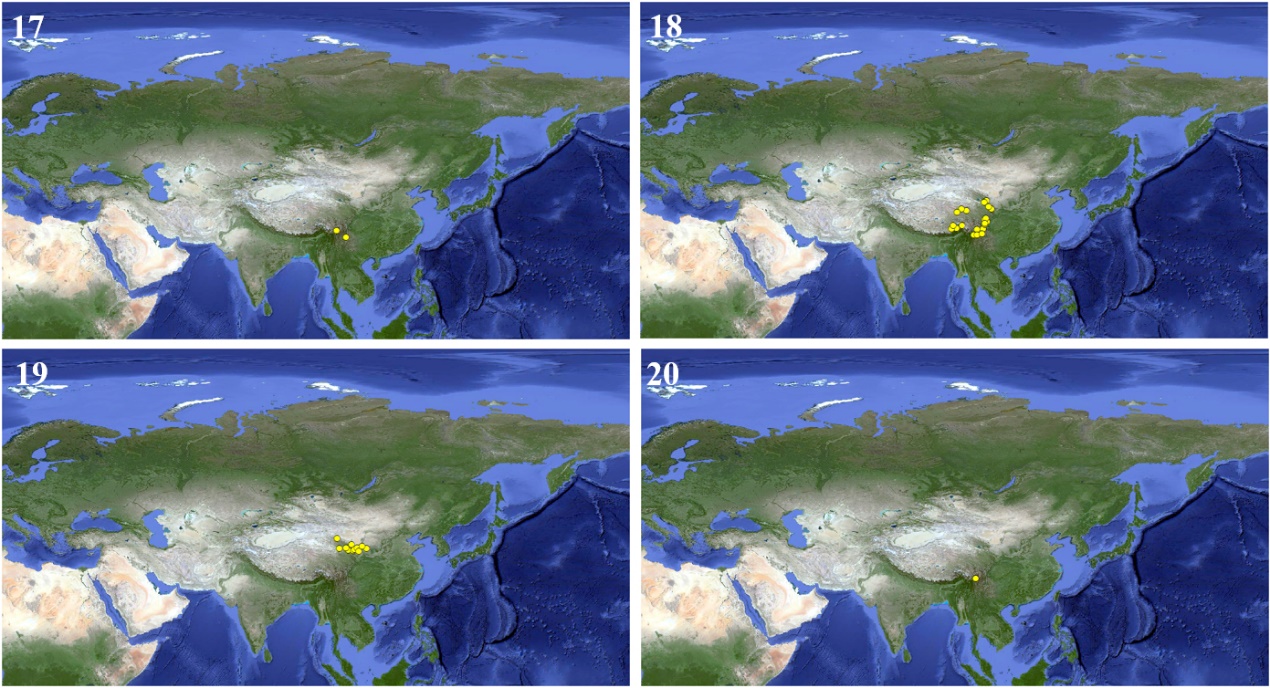

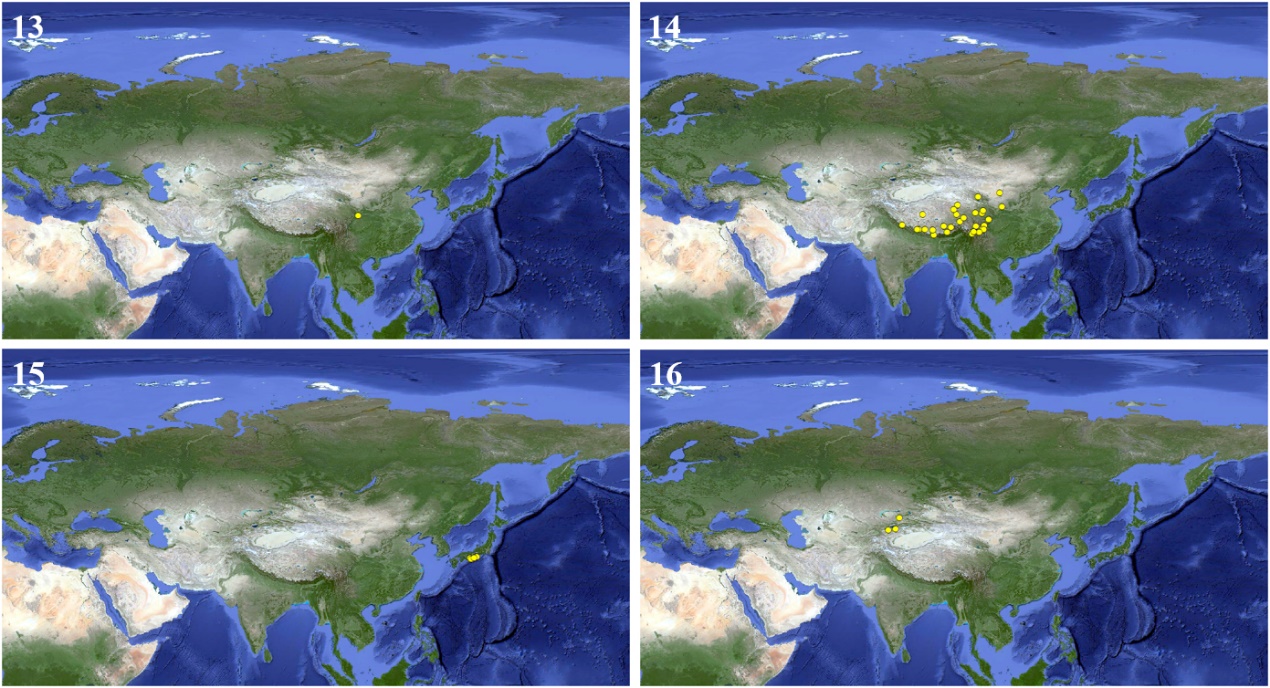

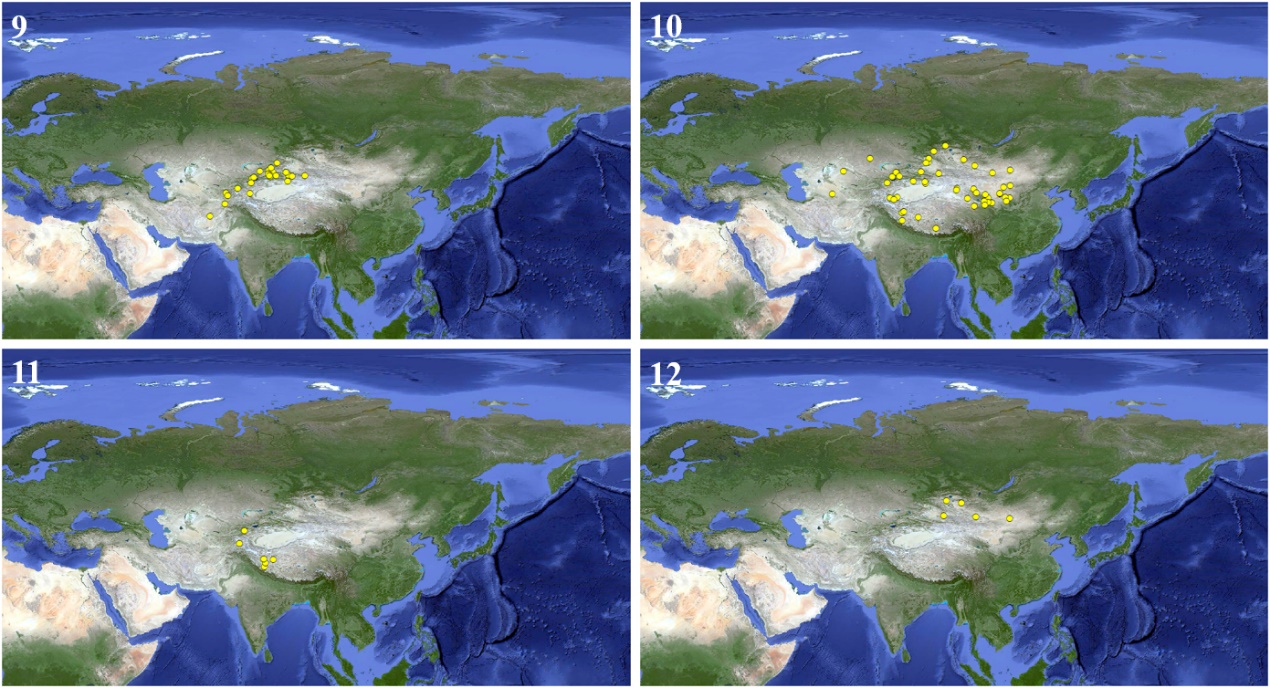


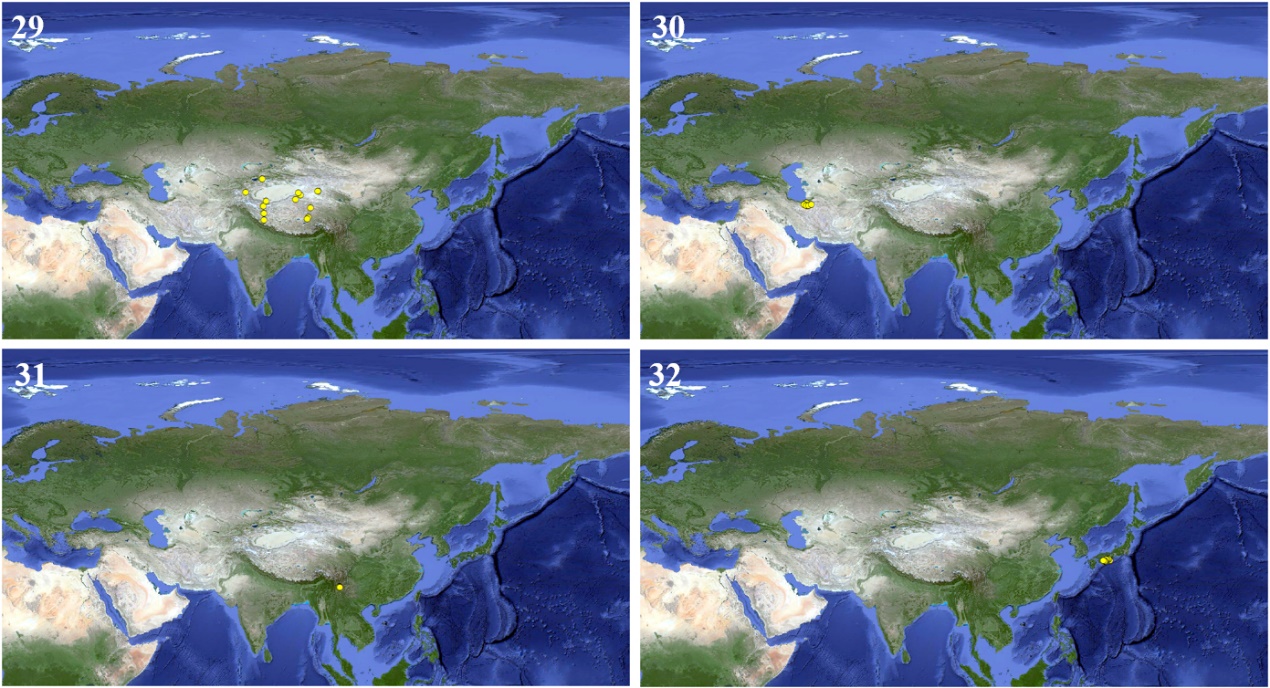

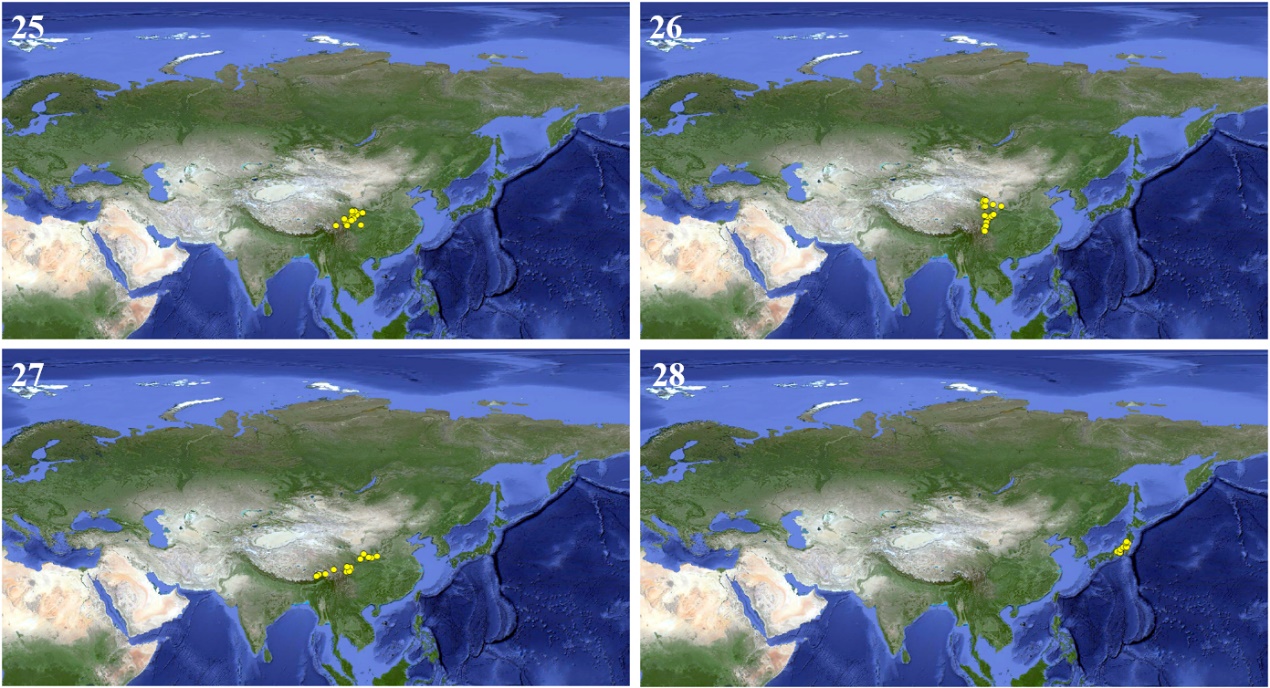

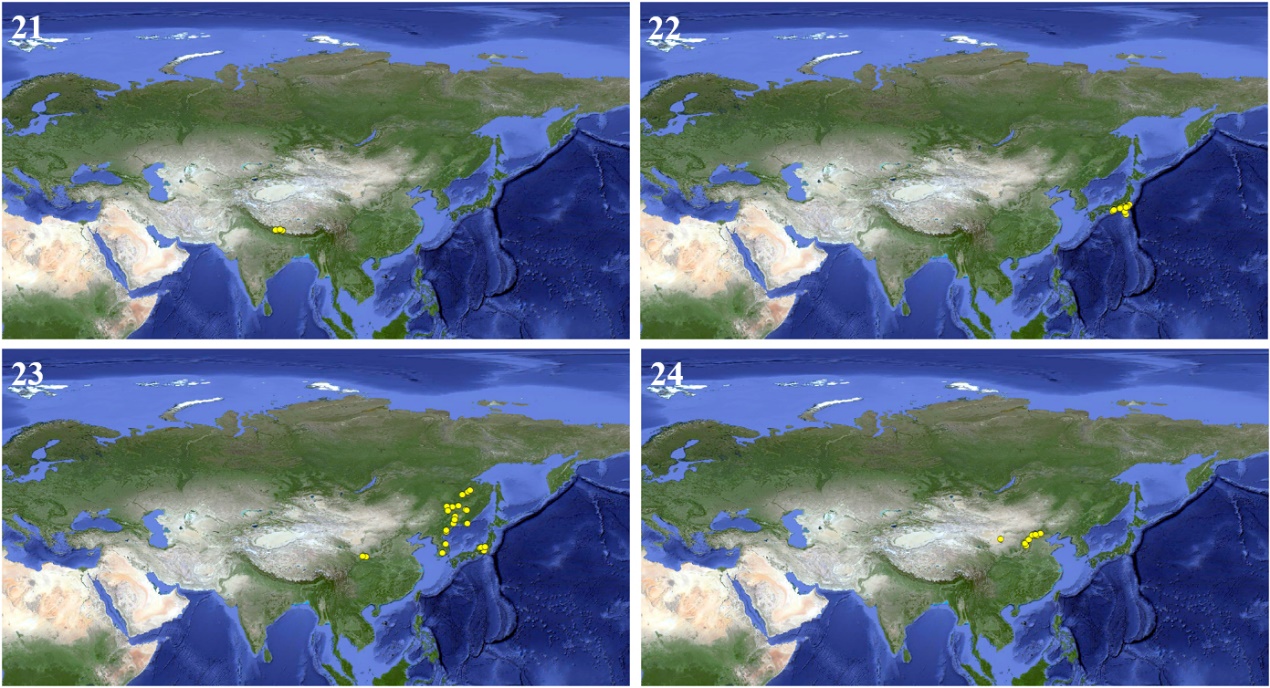


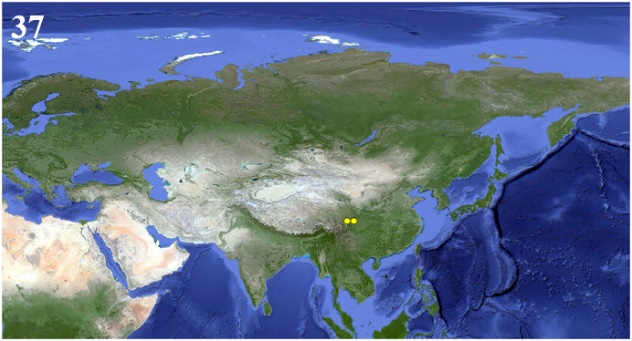

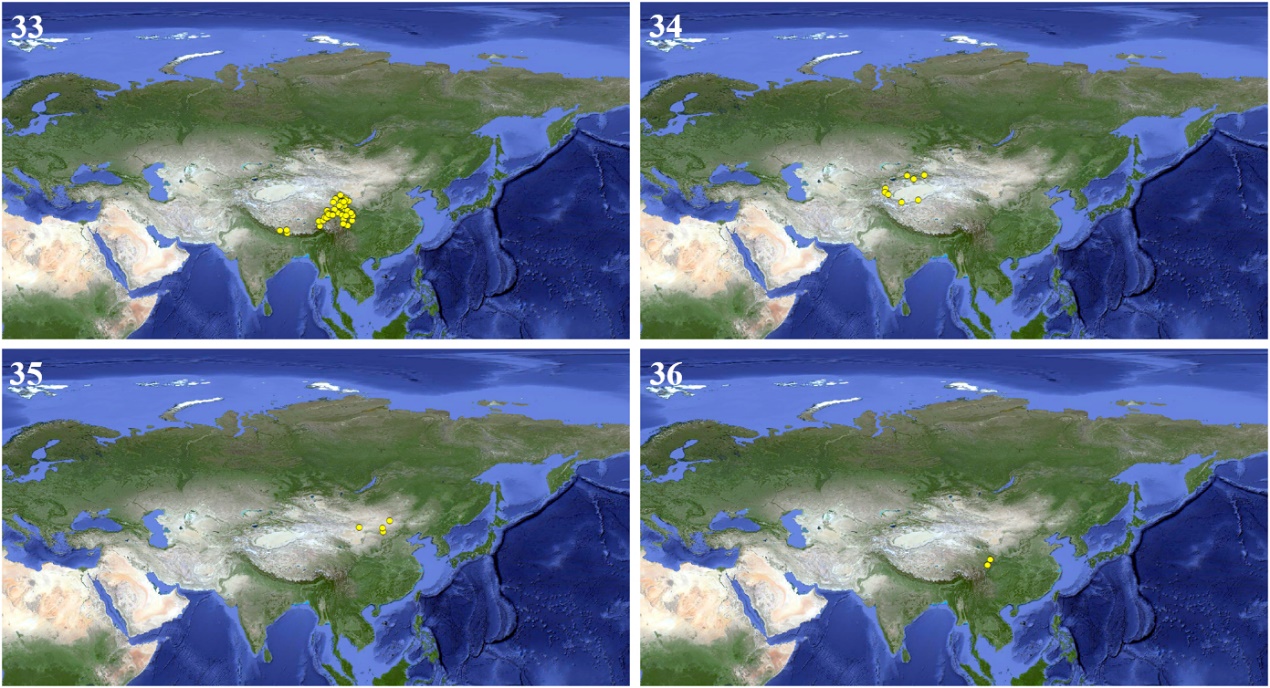


**Fig. S1** (b) Distributions of each species of *Ajania*, the species numbers corresponded to Table S2


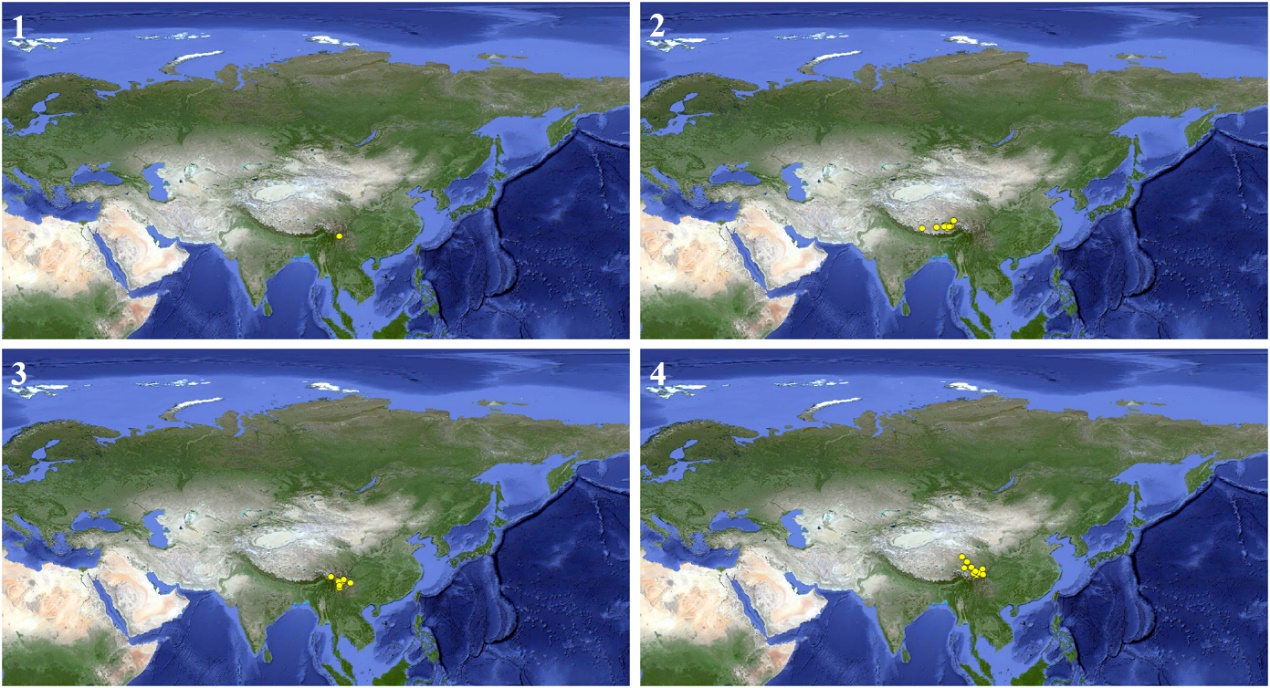


**
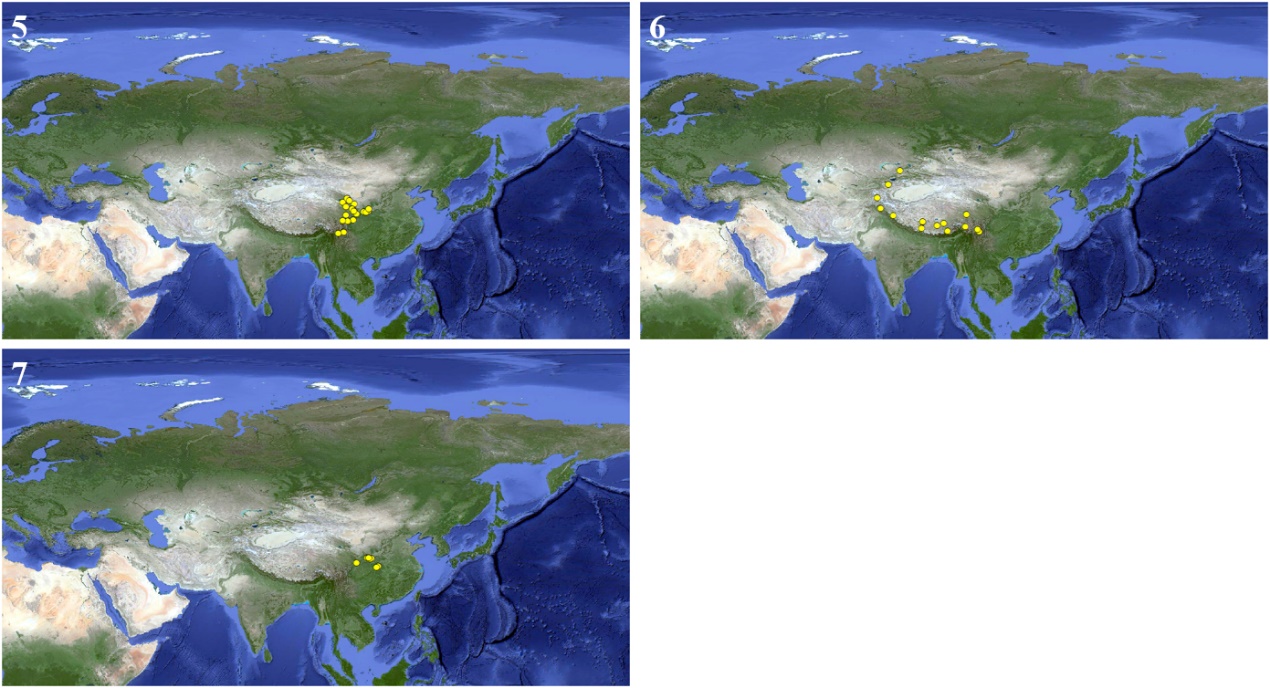
F****ig. S1 (c)** Distributions of each species of *Phaeostigma*, the species numbers corresponded to Table S2


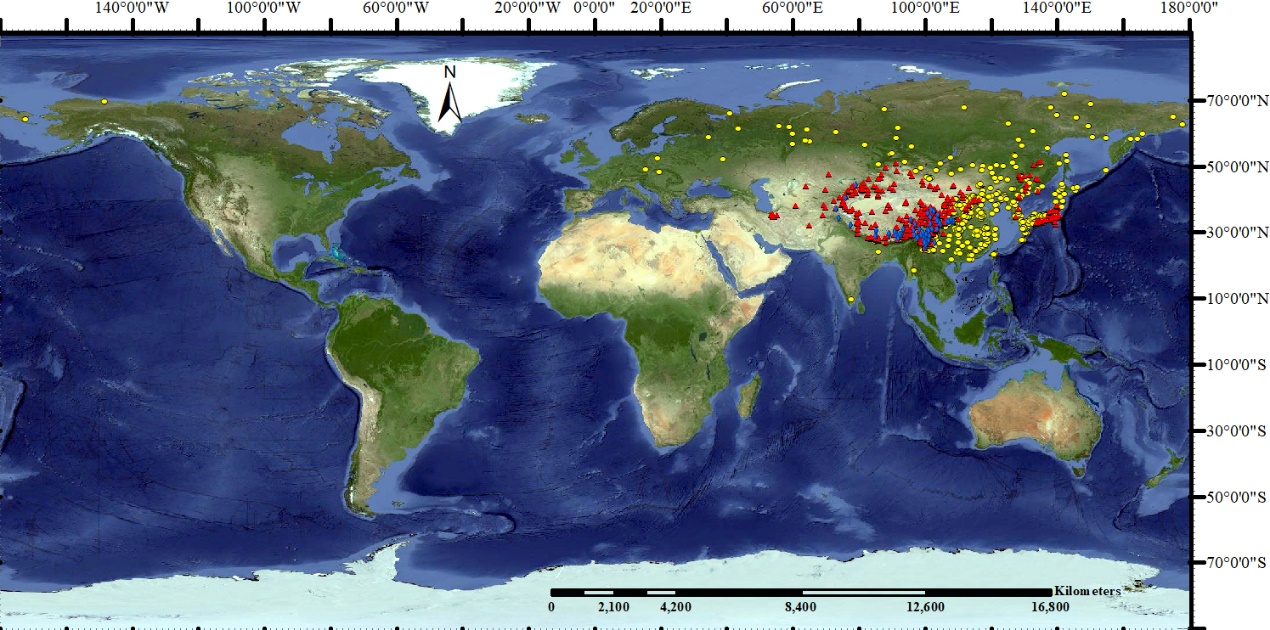


**Fig. S2** Distributions of Chrysanthemum (yellow), Ajania (red), and Phaeostigma (blue) based on GIS.


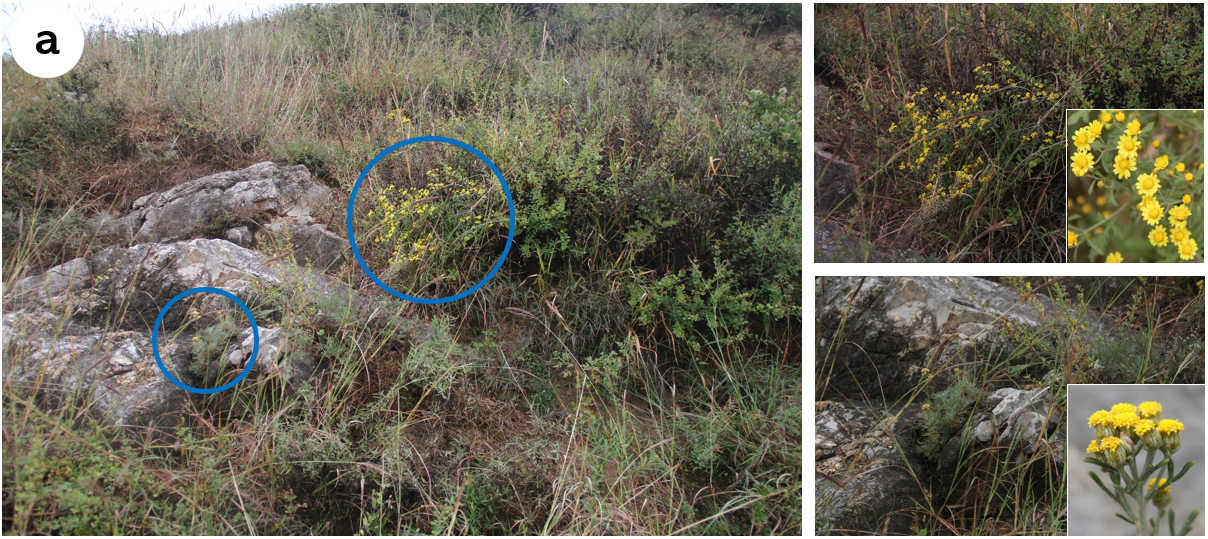


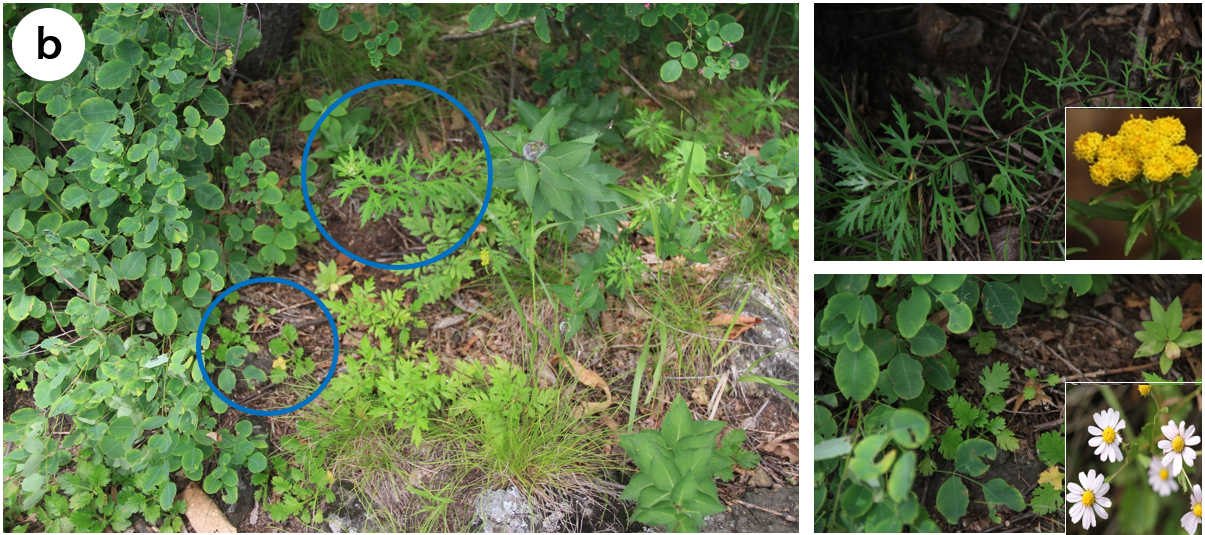


**Fig. S3** Close crossed distribution between (a) two groups of *Chrysanthemum* (blue), and (b) different species of *Chrysanthemum* and *Ajania* (blue). (a)*C. naktongense* and *C. lavandulifolium* in Zhangjiakou, Hebei Province. (b)*A. pallasiana* and *C. naktongense* in Yichun, Heilongjiang Province. Right: Partial enlarged view and capitula of related species (capitula of *A. pallasiana* from *PPBC*).


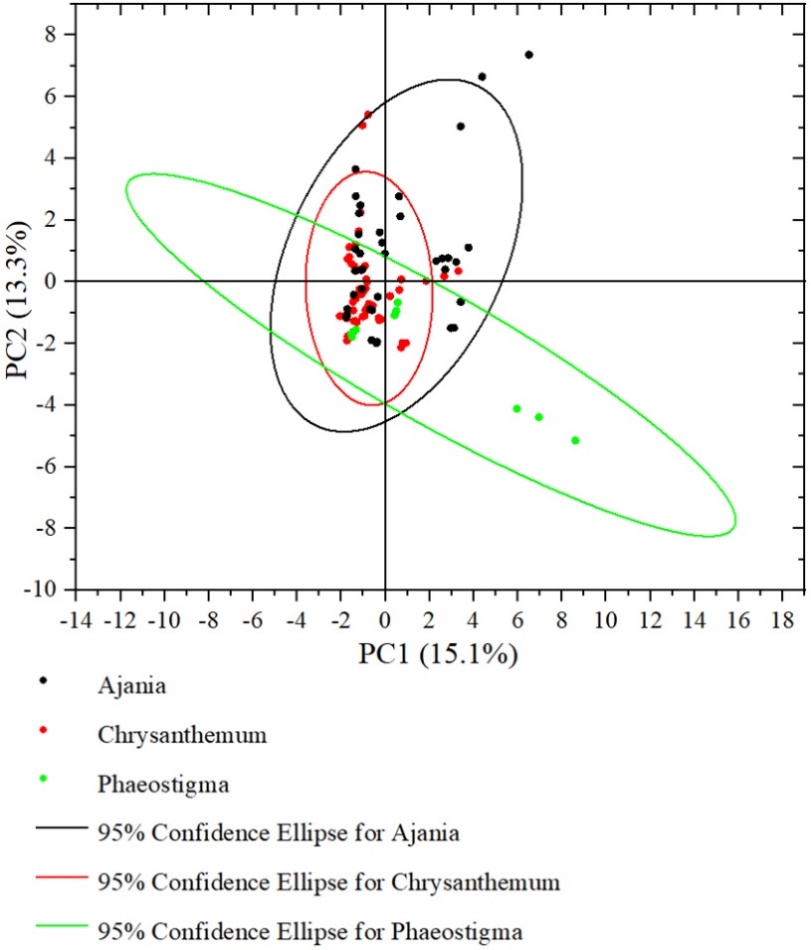


**Fig. S4** PCA of *Chrysanthemum*, *Ajania*, and *Phaeostigma* based on 30 secondary metabolites.


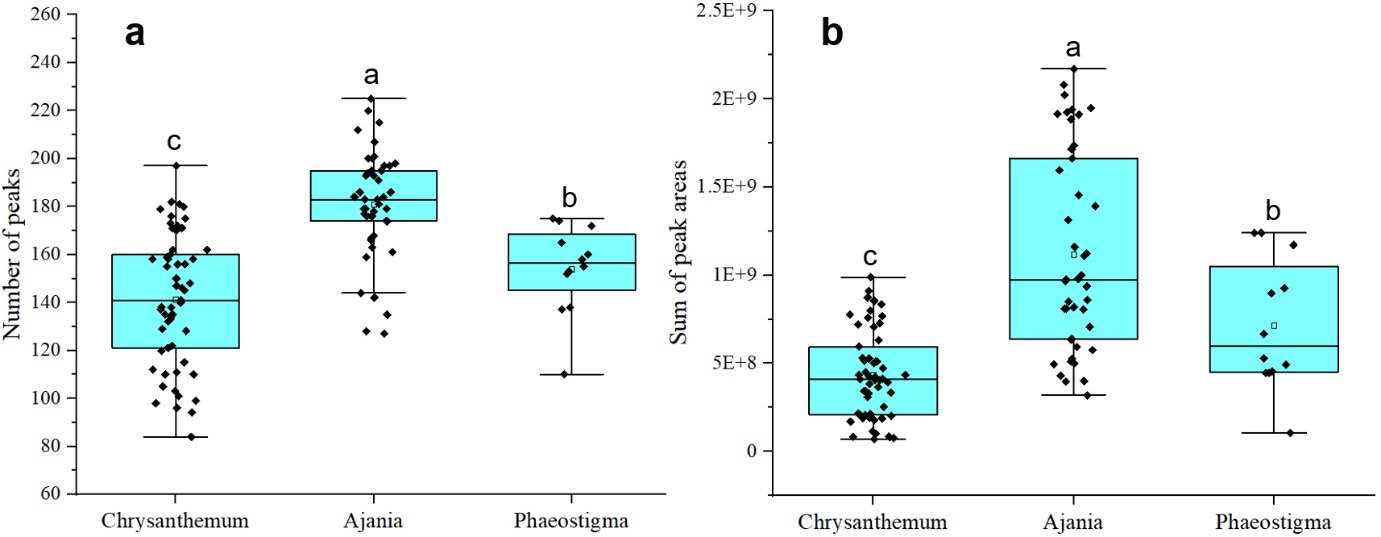


**Fig. S5** (a) Number of peaks and (b) sum of peak areas based on GC-MS of *Chrysanthemum*, *Ajania* and *Phaeostigma* (*P*＜0.05)


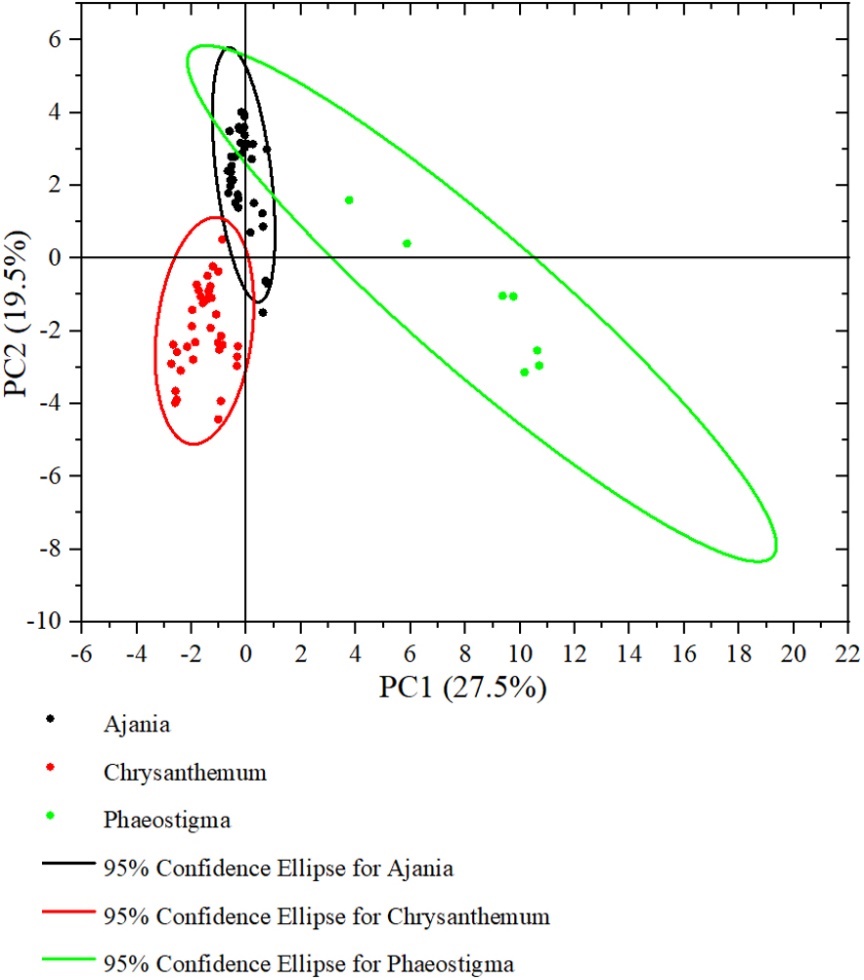


**Fig. S6** Principal component analysis (PCA) and 95% confidence of *Chrysanthemum*, *Ajania*, and *Phaeostigma* based on 31 traits.


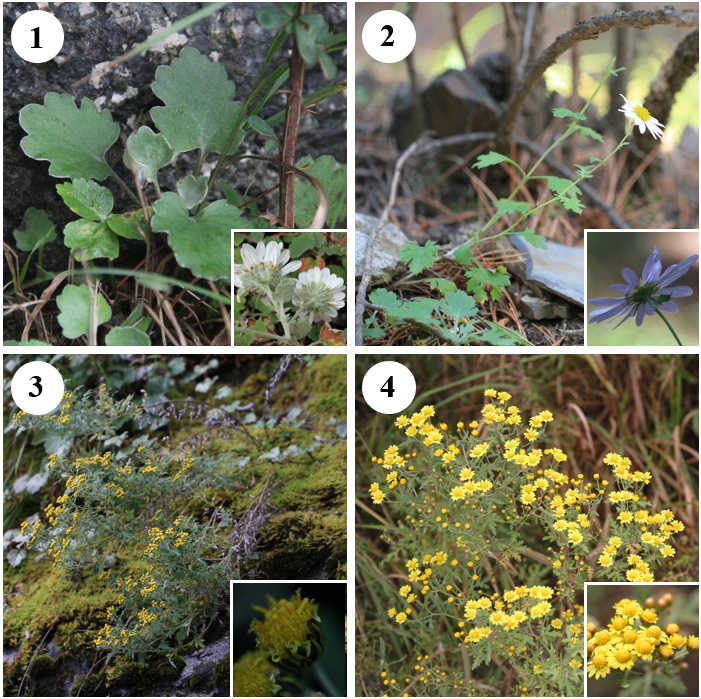


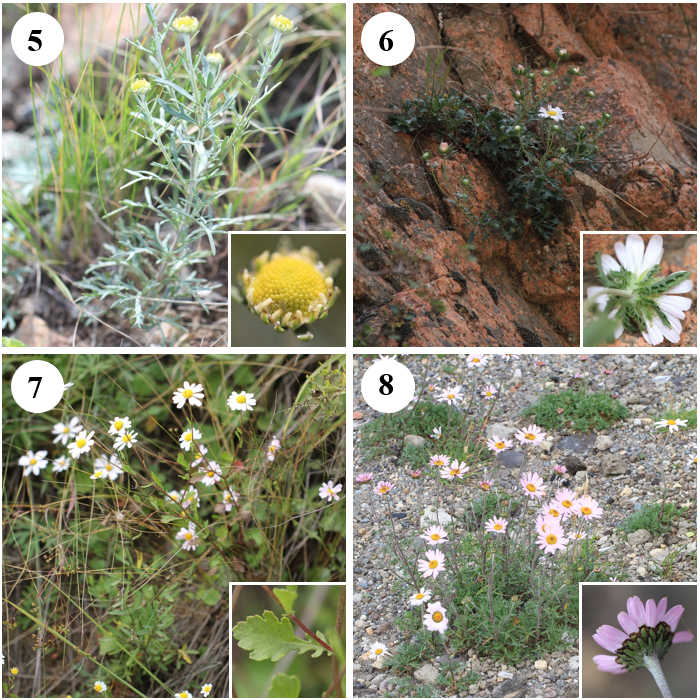


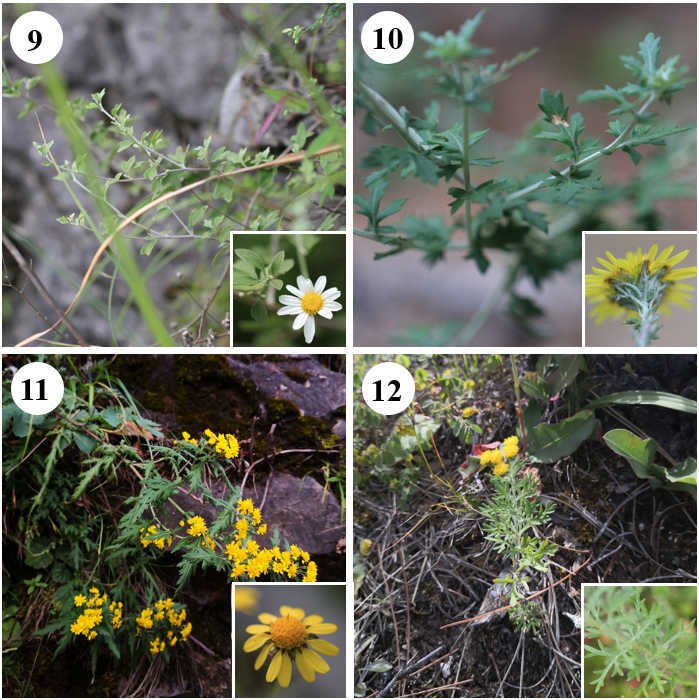


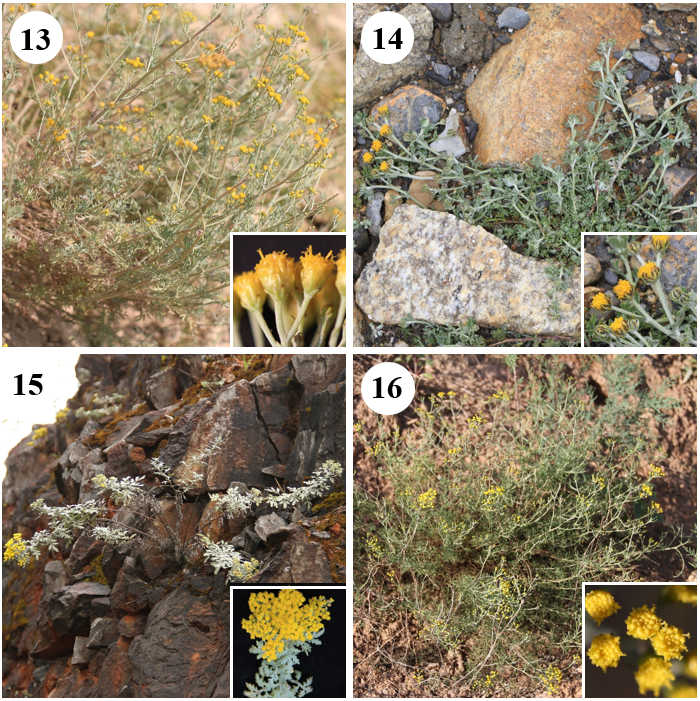


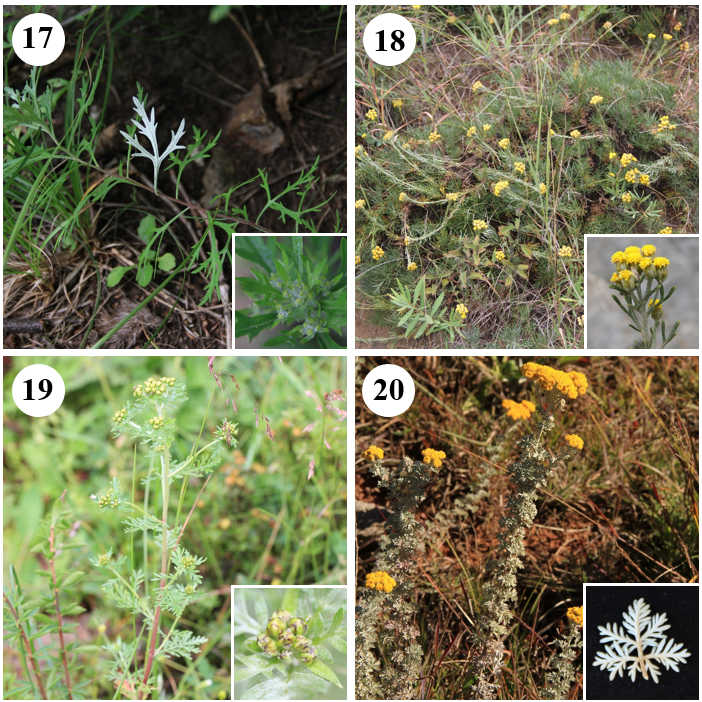


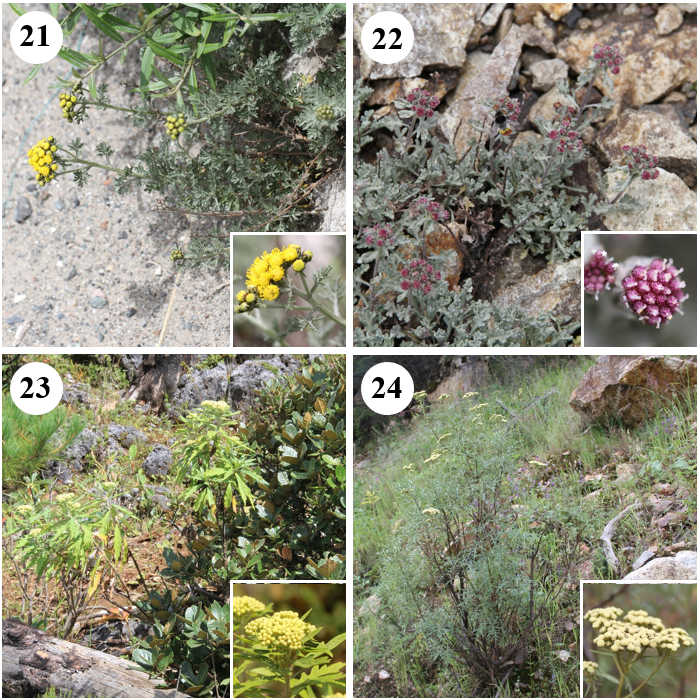


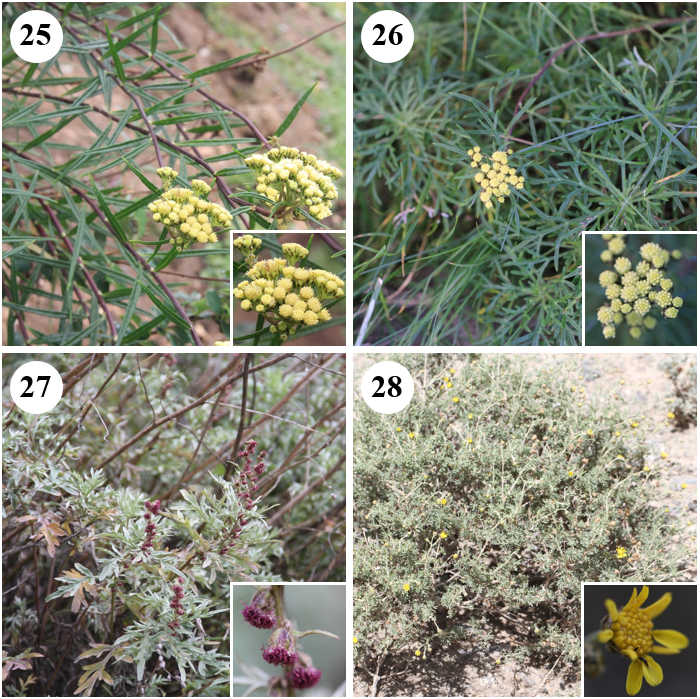


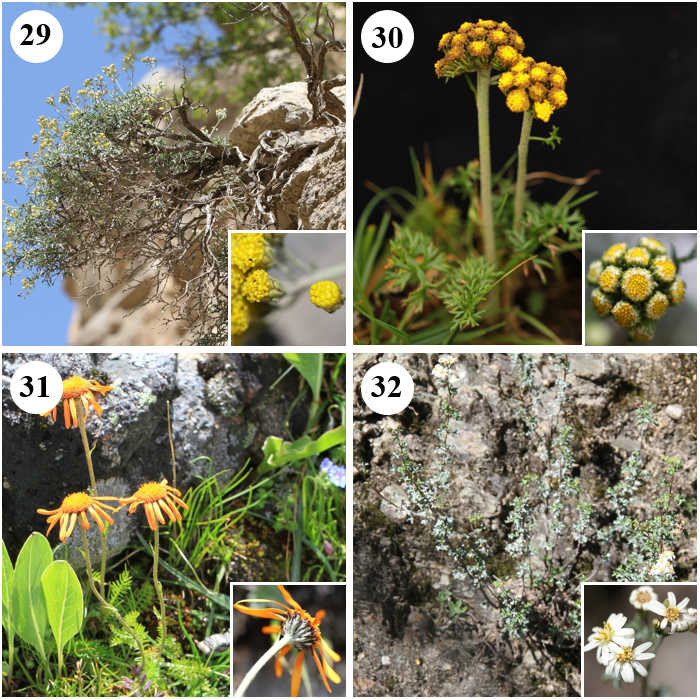


**Fig. S7** Field phenotype of *Chrysanthemum* species and its allies based on field investigation.

1 *C. argyrophyllum*; 2 *C. chanetii*; 3 C*. glabriusculum*; 4 *C. lavandulifolium*; 5 *C. maximowiczii*; 6 *C. mongolicum*; 7 *C. naktongense*; 8 *C. oreastrum*; 9 *C. rhombifolium*; 10 *C. foliaceum*; 11 *C. seticuspe*; 12 *A. adenantha*; 13 *A. fastigiata*; 14 *A. khartensis*; 15 *A. myriantha*; 16 *A. nematoloba*; 17 *A. pallasiana*; 18 *A. parviflora*; 19 *A. przewalskii*; 20 *A. sericea*; 21 *A. tenuifolia*; 22 *P. purpureum*; 23 *P. quercifolium*; 24 *P. ramosum*; 25 *P. salicifolium*; 26 *P. variifolium*; 27 *Ar. speciosa*; 28 *B. pulvinatum*; 29 *H. alashanensis*; 30 *H. delavayi*; 31 *T. tatsienense*; 32 *F. mekongensis*. Images only show the phenotype of species, not the size.


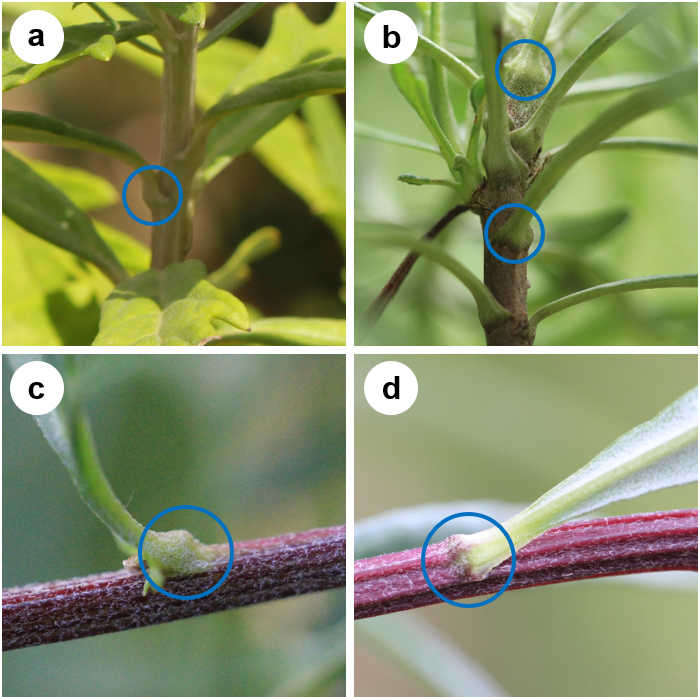


**Fig. S8** Lateral expansion of petiole base of *Phaeostigma*: (a) *P. quercifolium,* (b) *P. ramosum*, (c) *P. variifolium*, and (d) *P. salicifolium*.


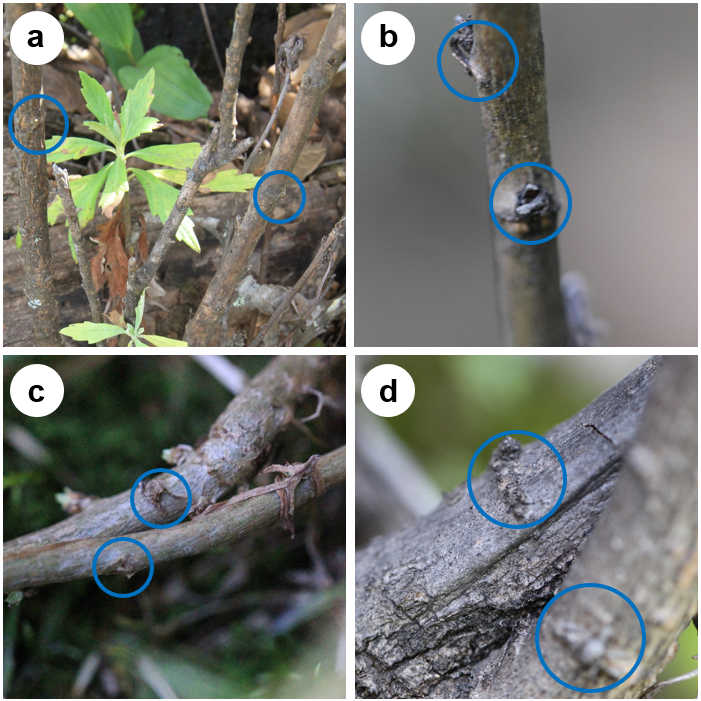


**Fig. S9** The leaf scar of *Phaeostigma*: (a) *P. quercifolium*, (b) *P. ramosum*, (c) *P. variifolium*, and (d) *P. salicifolium*.


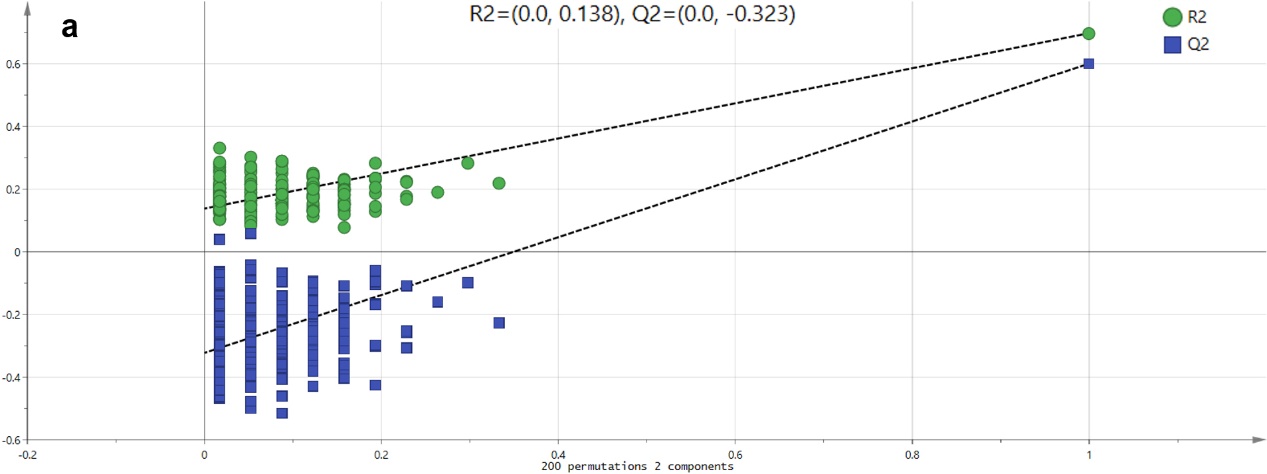


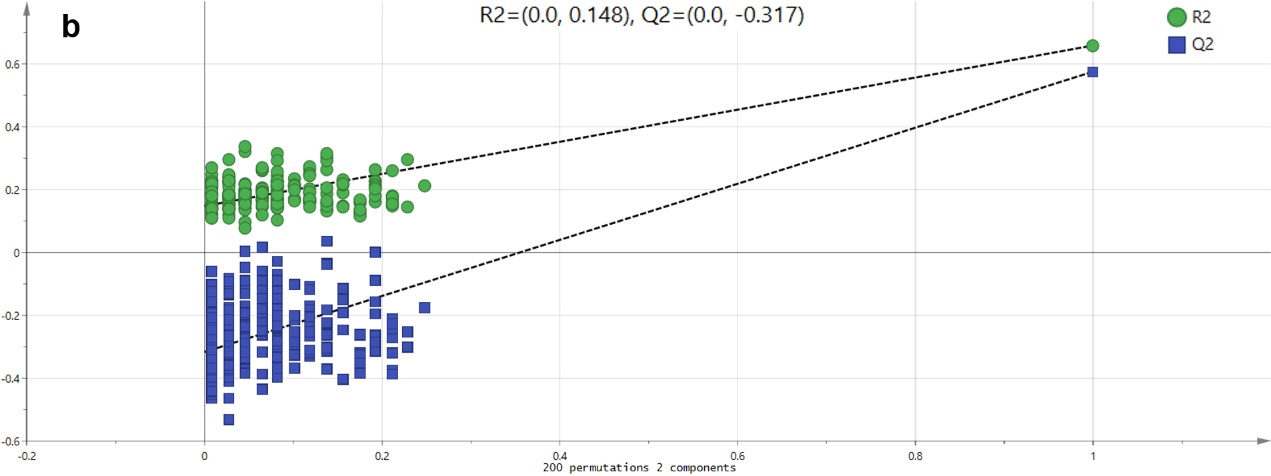


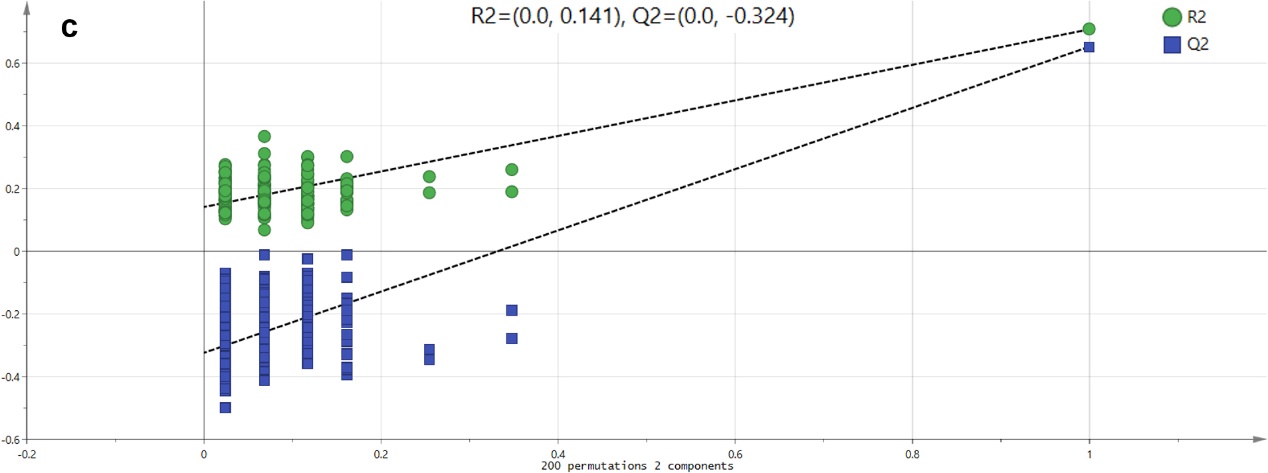


**Fig. S10** Permutations for OPLS-DA model. Selected (a*) Chrysanthemum*, (b) *Ajania*, and (b) *Phaeostigma* as variables.
